# The Effect of Perspective on Presence and Space Perception

**DOI:** 10.1371/journal.pone.0078513

**Published:** 2013-11-06

**Authors:** Yun Ling, Harold T. Nefs, Willem-Paul Brinkman, Chao Qu, Ingrid Heynderickx

**Affiliations:** 1 Interactive Intelligence Group, Delft University of Technology, Delft, The Netherlands; 2 Philips Research Laboratories, Eindhoven, The Netherlands; ICREA-University of Barcelona, Spain

## Abstract

In this paper we report two experiments in which the effect of perspective projection on presence and space perception was investigated. In Experiment 1, participants were asked to score a presence questionnaire when looking at a virtual classroom. We manipulated the vantage point, the viewing mode (binocular versus monocular viewing), the display device/screen size (projector versus TV) and the center of projection. At the end of each session of Experiment 1, participants were asked to set their preferred center of projection such that the image seemed most natural to them. In Experiment 2, participants were asked to draw a floor plan of the virtual classroom. The results show that field of view, viewing mode, the center of projection and display all significantly affect presence and the perceived layout of the virtual environment. We found a significant linear relationship between presence and perceived layout of the virtual classroom, and between the preferred center of projection and perceived layout. The results indicate that the way in which virtual worlds are presented is critical for the level of experienced presence. The results also suggest that people ignore veridicality and they experience a higher level of presence while viewing elongated virtual environments compared to viewing the original intended shape.

## Introduction

Presence is defined as the sense of being in one place or environment, when one is physically situated in another [1]. A high level of presence is thought to be beneficial for various types of virtual reality applications, such as virtual reality exposure therapy [2], training and education [3], and entertainment [4]. In the current paper, we investigate how presence is affected by various aspects of perspective when looking into a virtual world ‘through’ a screen.

**Figure 1:**
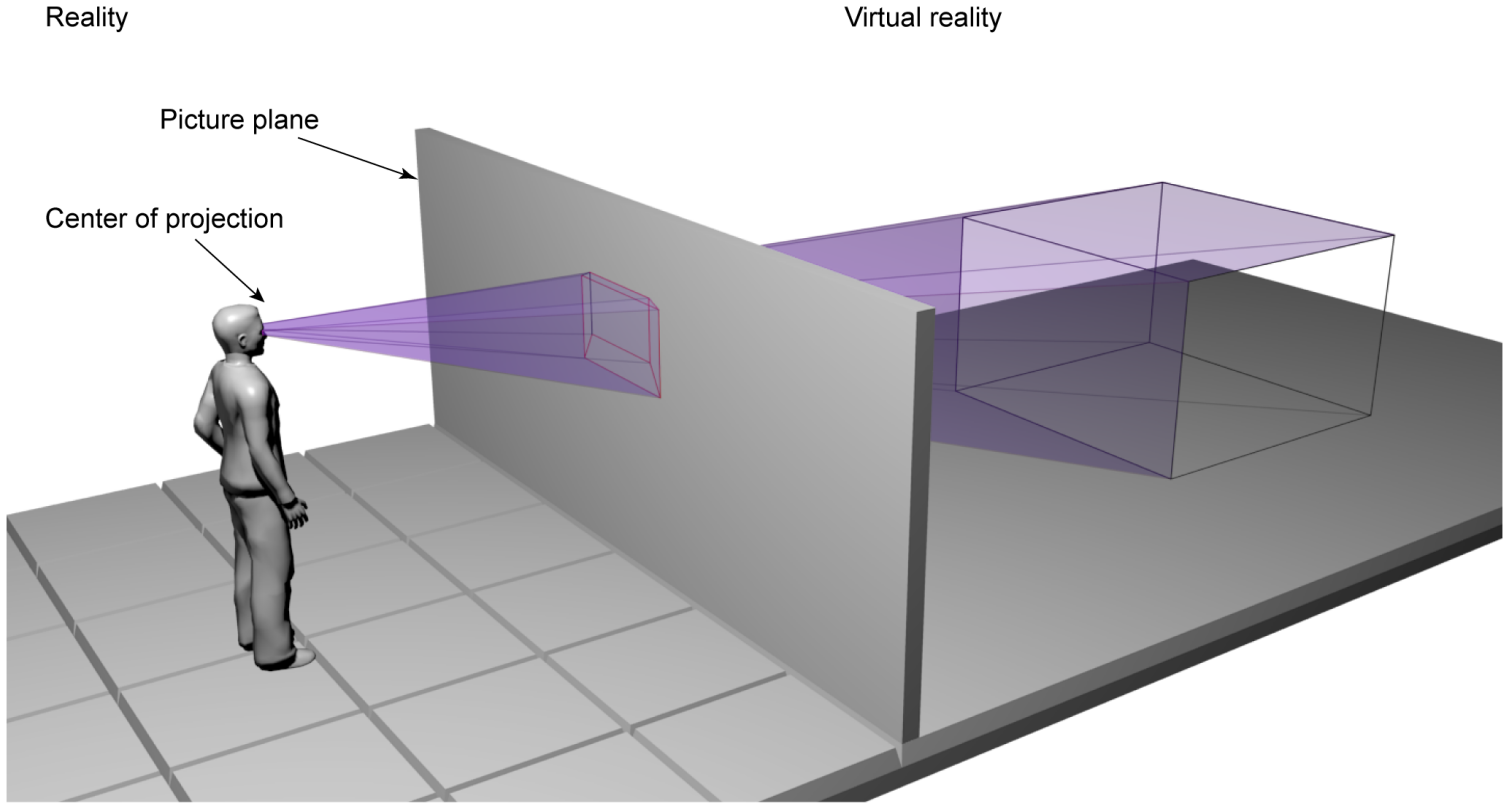
The ‘Alberti window’: viewing a virtual reality environment from the center of projection shows the viewer an image as if the viewer looks at the scene through the frame of the screen.

All perspective images have a center of projection (*CoP*). A perspective projection creates 2D images of 3D objects by projecting lines from a *CoP* through a picture plane until they meet the objects. The position from which the observer looks at the virtual world is his/her vantage point (only monocular observation or a cyclopean eye for binocular observation will be considered). When the vantage point coincides with the *CoP*, the image in the eye is exactly the same as if the viewer looks at the scene through the frame of the screen (i.e., an ‘Alberti window’, see Figure 1). If the vantage point does not coincide with the *CoP*, the image in the eye is not the same as what the ‘Alberti window’ would provide.

What are the effects of moving the vantage point on the perceived spatial layout of a scene? Geometrical analyses suggest that displacements of the vantage point are associated with transformations of the structure of the 3D scene. Displacements of the vantage point orthogonally away from the *CoP* correspond to compressions (when moving the vantage point towards the screen, i.e., *CoP* behind the vantage point) and dilations (when moving the vantage point away from the screen, i.e., *CoP* in front of the vantage point). Displacements of the vantage point parallel to the display screen correspond to lateral shears of the intended scene in the direction opposite to the displacement direction [5]–[7].

Earlier empirical studies have investigated the effect of viewing an image from different vantage points on the perceived virtual space. Rogers [8] showed that displacements of the vantage point tend to induce perceptual changes similar to the transformations predicted by geometrical analyses. Later studies, however, showed that the perceived effect of a displacement of the vantage point was smaller than what was predicted from geometrical analyses [9]–[11]. Also Vishwanath et al. [12] reported that the effect of a displacement of the vantage point on the perception of geometrical objects was relatively small. Some authors even suggested that the transformations predicted by geometrical analyses were not what people perceived. When information from the picture surface was available, the human visual system was able to compensate for observing from a wrong vantage point. The compensation process recovered the correct *CoP* and reconstructed the appropriate spatial layout as it would be seen from the correct vantage point [13]–[15]. The latter statement, however, is not shared by all research; [9] showed that many shapes in an image looked distorted, even when observed from the correct vantage point. In addition, Kellner et al. [16] found that even the correctly calibrated *CoP* could not entirely compensate for the distance underestimation effects in an immersive virtual environment.

The way the virtual environment is perceived may affect the experienced level of presence. Hendrix and Barfield [17] showed that moving the *CoP* from behind the vantage point forward to the vantage point improved the level of experienced presence; note that they did not test the condition in which the *CoP* was in front of the vantage point. To find ways to improve presence, we manipulate in our current research the level of displacement of *CoP* orthogonally or laterally away from the vantage point and test whether there is a difference in presence and perceived spatial layout of the virtual environment. We hypothesize that the level of presence increases (H1.1) and the perceived distortion of the virtual environment decreases (H1.2) when the *CoP* moves closer to the vantage point. To test whether people report a higher level of presence for their own preferred displayed scene, participants were asked to set their preferred *CoP* for various vantage points. We expect that a person's preferred *CoP* equals his/her vantage point (H1.3). Since so far, to our knowledge, there is no study into the relationship between perceived shape of a virtual environment and the sense of presence by manipulating *CoP*, we also looked at this relationship and hypothesize that the sense of presence can be predicted from the perceived layout of the virtual environment (H1.4).

In this study, the field of view (FOV) refers to the angle subtended from the eye to the left and right edge of the display screen, i.e., horizontal field of view. This angle is limited by the physical display width, and can only be increased by replacing the display hardware with a larger size screen or moving the user physically closer to the display. A larger FOV evokes a higher level of presence even with the same pre-recorded virtual trajectory when the vantage point is behind the *CoP* [18]. When the vantage point coincides with the *CoP* and at a constant viewing distance, a larger screen size enables less distraction from outside and can display more information, and as such, improves the performance in a navigation task [19]. Cherni et al. [20] conclude that increasing the size of the screen, and so the FOV, improves perception of visual information and task performance in a virtual supermarket. Hence, all literature points towards a higher level of presence for a larger FOV. Most of these studies, however, varied the FOV by varying the display screen size, whereas in our experiment we change the FOV by manipulating the vantage point and we test its effect on presence and perceived shape for the condition in which the *CoP* is at the vantage point. Thus, based on the literature, we formulate our second hypothesis as: When the *CoP* is at the vantage point, a larger FOV increases the level of presence (H2.1) and does not affect the perceived shape of the virtual classroom (H2.2).

The human visual system extracts depth information from features in our visual field, such as occlusion and texture gradients, and from assumptions based on personal experience, such as height in the visual field and relative size [21]. Depth cues, such as stereovision, accommodation, and motion parallax may conflict with the perspective cue, because stereovision on a 2D display let us perceive an image as flat with every object positioned on the screen, while the perspective cue induces perceived depth with objects at different distances. The conflicting binocular information may suppress the perceived depth from monocular cues resulting in a flattening of the perceived depth. However, viewing with one eye can eliminate the conflicting disparity cue, making the depth perception more consistent with the monocular cues [22], [23]. Earlier studies also showed that viewing 2D pictures with one eye yielded a larger depth gain [24] and a better impression of depth [25] compared to viewing with two eyes. Therefore, we hypothesize that monocular viewing results in a higher level of presence (H3.1) and more depth impression (H3.2) than binocular viewing on a 2D display (H3).

To also investigate the effect of screen size, we selected a projector display, typically used in workplace environments, and a TV screen, typically used in the homes, and placed them at different distances in order to keep the FOV constant. The degree of cue conflicts in perceived depth varies with the viewing distance and viewing mode [21], [26]; that is the cue conflict between perspective and stereovision on a 2D display is stronger when the viewer is sitting closer to the screen. When manipulating screen size and viewing distance such that the FOV remains constant, it has been shown that a smaller screen size, and so, a shorter viewing distance, results in a worse comprehension of distance in a manipulation task [27]. Additional cues which signal a flat screen (e.g., stereovision, accommodation, etc.) are more apparent for a small size screen (TV) at a closer viewing distance than for a larger size screen (projector) at a larger viewing distance. Therefore, we hypothesize that for the same content, the depth perception on the TV is flattened with respect to the depth perception on the projector. As a consequence, we expect the perceived presence to be larger on the projector than on the TV. Hence, the related hypotheses are that, when the *CoP* is at the vantage point, individuals report higher levels of presence (H4.1.1) and a more stretched depth (H4.1.2) on the projector displaying a life-size virtual world than on the TV displaying the same content with the same FOV (H4.1).

**Figure 2:**
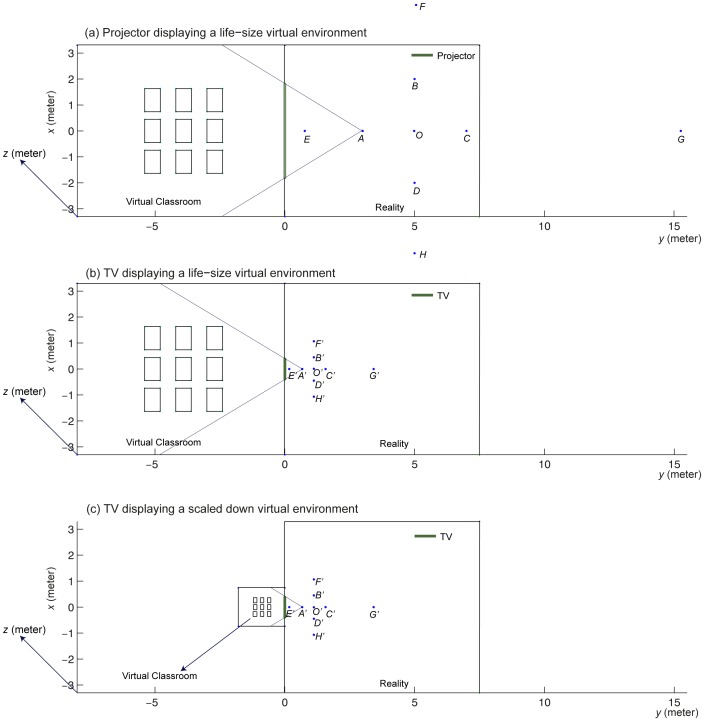
Projector (a) and TV (b) showing a life-size virtual classroom, and TV showing a scaled-down virtual classroom (c). Four vantage points *A*, *B*, *C* and *D* were used for the experiment.

Displaying exactly the same content on a large-size projector and on a small-size TV implies that the content on the TV is scaled down with respect to the content on the projector. Therefore, we refer to this condition as “scaled-down” virtual reality. Alternatively, when the *CoP* is at the vantage point, the FOV is kept constant for both a large and small screen, and they both display real-size virtual reality (as if looking through a real window), the projector shows a larger part of the virtual classroom than the TV (see Figure 2). Ni et al. [19] showed that at a constant viewing distance, a larger screen size can display more information and improves the performance in a virtual environment. As earlier studies also suggest that looking at a 2D picture from a larger distance can increase the illusion of depth perceived from 2D pictures [22], [23], we expect that individuals report higher levels of presence when exposed to a projector displaying a life-size virtual world than when exposed to a TV displaying a life-size virtual world when the *CoP* is at the vantage point (H4.2).

Now it is still unclear whether for smaller screen displays such as a TV, life-size virtual reality is preferable over scaled-down virtual reality. A TV displaying life-size virtual reality is more real, and therefore probably would lead to a higher level of presence than the scaled-down scene, but it only can show a limited part of the virtual world (see Figure 2b). A TV displaying scaled-down virtual reality is less real (since all parts of the scene do not have a realistic size), but it can display a larger part of the virtual scene, and so, may include more depth cues like perspective lines and size differences (see Figure 2c). Hence, we assume that both effects compensate each other, and we propose for the hypothesis considering the difference between different TV display settings: When *CoP* is at the vantage point, individuals experience similar levels of presence on the TV when it displays a life-size virtual world or a scaled-down virtual world with the same FOV (H4.3).

The whole study comprised two experiments. The relationship between the various hypotheses and the experiments is summarized in Table S1. In both experiments, a virtual classroom being a replication of a real experimental room was shown to the participants. The first experiment consisted of three sessions with three different display settings: a projector displaying a life-size virtual classroom, a TV displaying a life-size virtual classroom and a TV displaying a scaled-down virtual classroom. In each session, four vantage points were chosen, and the orthogonal or lateral displacement between the *CoP* and vantage point was manipulated. The participants were asked to report their feelings of presence of the conveyed virtual classroom. At the end of each session in experiment one, participants set their preferred *CoP* at four different vantage points. To investigate participants' perceived layout of the virtual classroom, the second experiment was conducted. It consisted of two sessions, i.e., a projector displaying a life-size virtual classroom and a TV displaying a scaled-down virtual classroom. The participants were asked to draw a floor plan of the virtual classroom.

## Experiment 1: presence

### Method

#### Ethics statement

All experiments were approved by the Delft University of Technology Human Research Ethics Committee. Written informed consent was obtained from all participants prior to the experiment. Each participant received a small gift for their contribution.

#### Participants

Twenty-four students, 8 females and 16 males, from the Delft University of Technology participated in the experiment. Their age ranged from 23 to 33 years with a mean of 26.15 (*SD* = 2.18) years. All participants were naive with respect to our hypotheses and were not involved in the setup of the experiments.

It is unclear to what extent the participants' experienced presence in the current study might have been affected by their previous experiences with virtual reality. Previous studies have found mixed results about the relationship between game playing experience and presence. Youngblut [28] and Romano and Brna [29] found a positive relationship, but Usoh [30] found a negative relationship. Ling et al. [31] found no significant correlation between game playing experience and experienced presence. Also Sylaiou et al. [32] found no statistically significant correlation between their participants' previous 3D computing experience and augmented presence. In the current study, most participants (i.e., twenty out of the twenty-four) had experienced virtual reality applications at least once before they took part in the experiment.

Since Ling et al. [31] found that people with better visual acuity experienced higher levels of presence than people with worse visual acuity, we measured the participants' visual acuity using ‘Freiburg Visual Acuity Test’ at a distance of 3 meters [33]. Participants viewed the virtual classroom with normal or corrected to normal visual acuity. Means and standard deviations of the participants' visual acuity (Snellen fraction) were 1.00 (0.30), 0.88 (0.29) and 1.16 (0.34) for left eye, right eye and both eyes, respectively.

#### Apparatus

The virtual classroom was displayed on a wall using a Panasonic PT-DX 500 projector and on a 37 inch Philips Cineos flat screen TV. The resolution of the projector was set to 1600*1200 (resulting at viewing point *A* (see Figure 2) in a visual angle of 0.0437°) and the screen size was 3.66 m in width and 2.75 m in height. The resolution of the TV was set to 1366*768 (resulting at the corresponding viewing point *A’* (see Figure 2) in a visual angle of 0.0026°) and the screen measured 0.82 m in width and 0.46 m in height. The horizontal field of view of both displays was kept equal by adapting the corresponding viewing distances (see for more details in the next paragraph). Also other display characteristics, such as brightness, contrast and color rendering, were made equal as well as possible. The projector was adjusted such that the bottom of the projected image coincided with the lower edge of the wall of the experimental room. The TV was placed on a table with a height of 0.94 m. The height of the *CoP* was adjusted to the height of each seated participant's eyes for all conditions within each session.

Four vantage points were chosen and they were labeled *A*, *B*, *C* and *D* in the projector condition and *A’*, *B’*, *C’* and *D’* in the TV conditions, as shown in Figure 2. The horizontal axis *x*, vertical axis *y* and the height of the space *z* are given in correct scales in Figure 2. Position (0, 0, z) refers to the screen center. In the projector life-size condition, the intended size of the virtual classroom was 6.6 m in width and 8 m in depth (see Figure 2a). Vantage points *A*, *B*, *C* and *D* were at (0, 3, *z*), (2, 5, *z*), (0, 7, *z*), (−2, 5, *z*) in meters, respectively. For each vantage point, the orthogonal or lateral displacements between the *CoP* and the vantage point were manipulated. For example, at vantage point *A* we showed the virtual classroom with the *CoP* at *C* and at *E* (0, 0.77, *z*), where the point *E* was chosen such that the ratio of the FOV at *E* over the FOV at *A* equaled 2.14, being also the ratio of the FOV at *A* (62.77°) over the FOV at *C* (29.30°). At vantage point *C*, we showed the classroom with the *CoP* at *A* and at *G* (0, 15.26, *z*), where *G* was selected based on the same ratio of 2.14. For vantage points *B* and *D*, the *CoPs* were chosen differently. At vantage point *B*, we chose a *CoP* at *O* (0, 5, *z*) being in front of the screen center and at *F* (4.76, 5, *z*) being 43.60° away from the screen center at twice the angle of *B* (21.80°). Similarly, at vantage point *D*, we selected two distorted *CoPs*, namely at *H* (-4.76, 5, *z*) and *O*.

In the TV life-size condition, the intended size of the virtual classroom was also 6.6 m in width and 8 m in depth (see Figure 2b). To keep the FOV the same as for the projector life-size condition, the four vantage points were chosen according to the ratio 4.46 between the projector's horizontal screen size (3.66 m) and the TV's horizontal screen size (0.82 m). This operation resulted in vantage points at *A’* (0, 0.67, *z*), *B’* (0.45, 1.12, *z*), *C’* (0, 1.57, *z*) and *D’* (-0.45, 1.12, *z*), again with all coordinates expressed in meters. In a similar way as for the projector, the corresponding points for the distorted *CoPs* were *E’* (0, 0.17, *z*), *G’* (0, 3.42, *z*), *F’* (1.07, 0, *z*), *H’* (−1.07, 0, *z*) and *O’* (0, 1.12, *z*).

When the TV displays exactly the same content as what the projector displays in life-size, every object displayed on the TV is physically much smaller than the same object displayed on the projector. As a consequence, the intended physical size of the classroom displayed on the TV in its “scaled-down” mode was 1.48 m in width and 1.79 m in depth (see Figure 2c). The four vantage points were the same as for the TV life-size condition, i.e., *A’* (0, 0.67, *z*), *B’* (0.45, 1.12, *z*), *C’* (0, 1.57, *z*) and *D’* (−0.45, 1.12, *z*).

**Figure 3:**
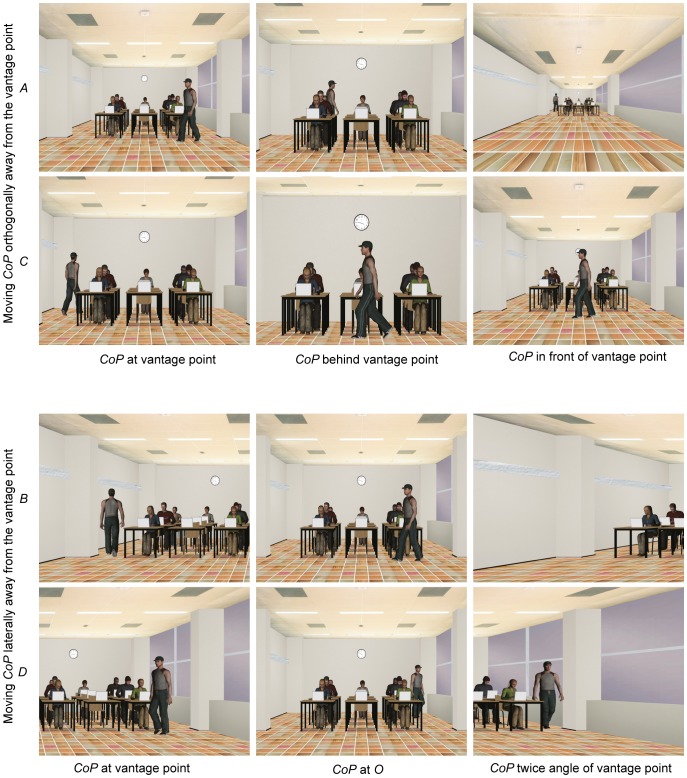
Snapshots of the displayed virtual classroom for the projector displaying life-size images. The four rows represent the four vantage points *A*, *C*, *B* and *D*, respectively. The three columns represent the three choices for the *CoP* at each vantage point.

The virtual world was made using WorldViz’s Vizard 3.0. Snapshots of the virtual classroom for the projector displaying the life-size virtual classroom are shown in Figure 3. In this virtual classroom, a person was walking around at a constant speed to induce more depth by changing his projected size.

#### Measurement

A modified version of an augmented reality presence questionnaire was used [34]. We chose for this particular presence questionnaire, since we were trying to show a virtual classroom that could be perceived as an extension of the real room. In the framework of augmented reality, this particular questionnaire already showed its merits in a virtual museum environment [32]. The standard questionnaire consisted of seven items which were rated on a seven-point Likert scale. The scores on the seven items were mapped onto three components, namely (1) Realness (how real the virtual classroom seems and how well it is integrated with the real room), (2) Spatial presence (degree of depth in the virtual classroom) and (3) Perceptual stress (whether the difference between the real and virtual room draws attention, and whether the perception of the virtual classroom needs some effort). The questions were adapted to match this particular virtual reality scene. For example, the question “Did the virtual objects appear to be (visualized) on a screen, or did you have the impression that they were located in space?” was modified to “Did the virtual classroom appear to be visualized on a screen, or did you have the impression that it was a room next door?’’. A general item that measured the sense of “an actual room being there” was added to the standard questionnaire. This general item was adopted from the Igroup presence questionnaire [35]. Most of the modifications of the standard augmented questionnaire consisted of replacing the term ‘virtual objects’ with the term ‘virtual classroom’. Questions that required more modification and the general item are given in Text S1. In the data analyses, only the total score of the whole questionnaire, ranging from 8 (i.e., a score of 1 on all 8 items) to 56 (i.e., a score of 7 on all 8 items), was used. The resulting presence questionnaire had good reliability with Cronbach’s *α* ranging from 0.92 to 0.98 across all 72 conditions.

#### Procedure

**Figure 4:**
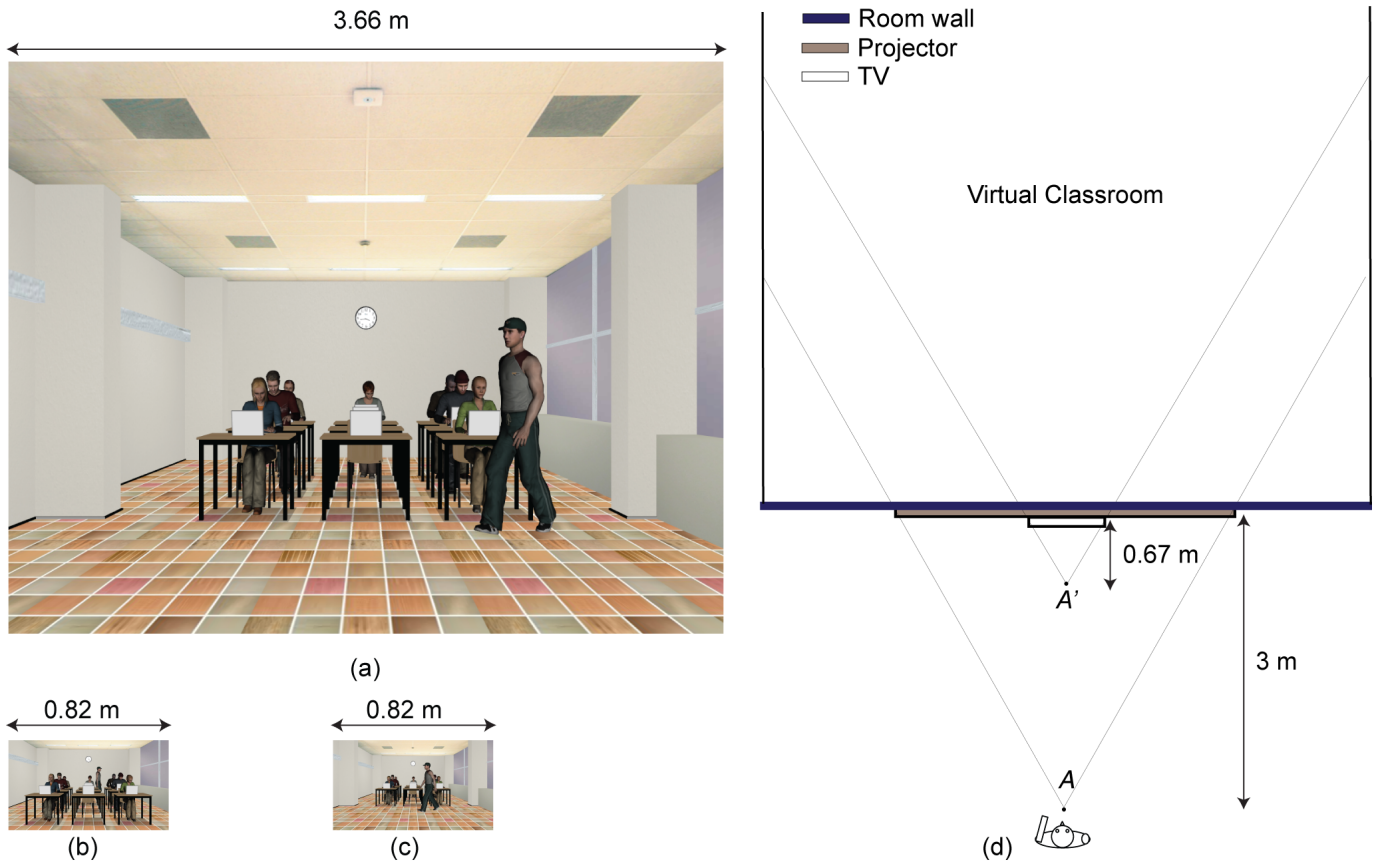
Differences between the three display settings. (a) Content of the projector displaying the life-size virtual classroom. (b) Content of the TV displaying the life-size virtual classroom. (c) Content of the TV displaying the scaled-down scene, and therefore, with the same content as for the projector. (d) Comparison of the relevant dimensions for the projector and the TV. Note that (a) (b) and (c) are scaled relatively to each other.

Prior to the first experiment, participants were provided with an information sheet, and the procedure was explained to them. They were then asked to sign an informed consent form. The experiment comprised three sessions with three different display settings: a projector displaying a life-size scene, a TV displaying a life-size scene and a TV displaying a scaled-down scene. Figure 4 shows an example of the differences between these three display settings. The order of the three sessions was counterbalanced among the participants. In each session, a virtual classroom with different *CoPs* was shown to the participants and they were asked to report their feelings of presence. The participants sat at four different vantage points in front of the displays. In total, the classroom was rendered with 12 different *CoPs* in each viewing condition. The participants were asked to view the 12 stimuli twice: once with one eye, and once with two eyes. Therefore, 24 conditions were assessed in each session. Note that in the remainder of the paper we will refer to stimuli when we discuss the 12 renderings for each display device, while we refer to conditions when we include the two viewing conditions. These 24 conditions were shown to each participant in a different random order, also different per session. To ensure proper one-eye viewing, participants wore an opaque glass to block the view of either their left or right eye according to their own preference. There was a debriefing session afterwards, in which the participants’ experiences were discussed and full details of the experiment were explained to them.

### Results

**Figure 5:**
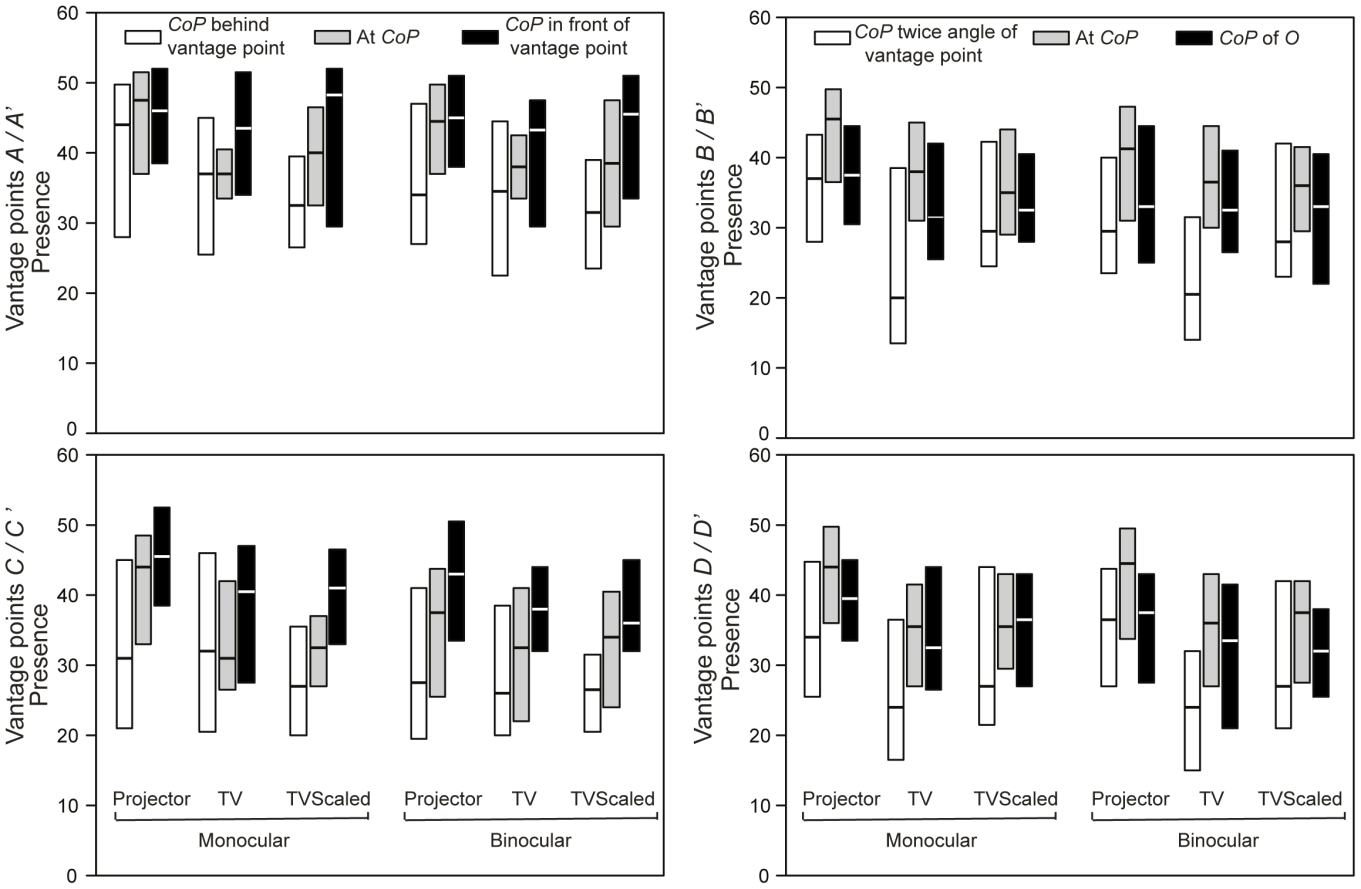
Medians and interquartile ranges for presence at four vantage points, with three levels of *CoP* and in two different viewing modes for all three display settings. The horizontal line represents median and the distance between the top edge and the bottom edge represents interquartile range.

Medians and interquartile ranges of the presence scores at four different vantage points, with three levels of distortion (i.e., with three different *CoPs*) and in two different viewing modes are shown for the three display settings in Figure 5. The horizontal axis consists of six categories, including the two viewing modes (monocular and binocular viewing) for the three different display settings. The presence scores for the three different *CoPs* are shown as different bars, whereas each of the graphs represents a vantage point. As some of the depended variables deviated from normality, non-parametric analyses were conducted, including Wilcoxon Signed Ranks tests for paired comparisons, Friedman tests for comparing several related groups, and factorial repeated-measures ANOVAs on aligned rank data for non-parametric factorial analyses [36]. Note that the effect size for Friedman test was calculated by using the significance value of the chi-square test statistic to find the associated value of *z* from a table of probability values for the normal distribution, and then use the conversion to *r* [37].

Given that only one virtual environment was used, there might be an order effect on presence, i.e., presence judgments in one trial may have influenced those of the subsequent trials. Medians and interquartile ranges (in brackets) were 39.25 (*13.38*), 34.75 (*18.0*) and 32.75 (*15.38*) for the first, the second and the third exposure session respectively. To test the order effect on presence, a Friedman test was conducted using median presence scores in the three exposure sessions, which resulted in no significant effect of order on presence (*χ^2^*(2, *N* = 24) = 0.96, *p*  = .62, *r = *.06).

A repeated-measures ANOVA on the aligned ranks data was first conducted using vantage point, viewing mode, *CoP* and display setting as independent factors, and presence as dependent variable to find the main and interaction effects. The results showed that vantage point (*F*(3,69) = 24.07, *p*<.001, *η* ^2 = ^.51), viewing mode (*F*(1,23) = 11.65, *p* = .002, *η* ^2^ = .34), *CoP* (*F*(2,46) = 30.08, *p*<.001, *η* ^2^ = .57) and display setting (*F*(2,46) = 6.78, *p* = .006, *η* ^2^ = .23) all affected the participants' experienced presence significantly. We also found a significant interaction between display setting and vantage point (*F*(6,138) = 2.76, *p* = .04, *η* ^2^ = 11), between *CoP* and vantage point (*F*(6,138) = 10.19, *p*<.001, *η* ^2^ = .31), and between display setting, *CoP* and vantage point (i.e., a three-way interaction) (*F*(12,276) = 4.53, *p* = .001, *η* ^2^ = .17). These interaction effects were mainly caused by the vantage point, due to the fact that the rendered perspective distortions at vantage points *A* and *C* (i.e., moving the *CoP* in front of or behind the vantage point) were different from the perspective distortions at vantage points *B* and *D* (i.e., moving the *CoP* to the left or right of the vantage point).

To test the hypotheses H1.1 (the effect of *CoP* on presence), H2.1 (the effect of FOV by manipulating vantage point on presence), H3.1 (the effect of viewing mode on presence) and H4.1.1, H4.2 and H4.3 (the effect of display settings on presence) more specifically, additional analyses were conducted as explained in the following separate subsections. Note that as the overall test found significant effects, no alpha correction was applied to the multiple tests in the follow-up analyses.

#### Vantage point

The presence scores per vantage point were calculated by taking the median score across the three display settings under binocular viewing when the *CoPs* coincided with the vantage points (note that this represents a more limited data set than what was used for the overall repeated-measures ANOVA). Medians and interquartile ranges (in brackets) of these scores were 38.5 (*12*), 37.5 (*12.25*), 34 (*14*), 38 (*11.9*) for *A*, *B*, *C* and *D,* respectively. To test the effect of vantage point on presence (H2.1), a Friedman test was done using vantage point as an independent variable and presence as the dependent variable. A significant effect of vantage point, and so FOV, on the level of experienced presence was found (*χ^2^*(3, *N* = 24) = 21.33, *p*<.001, *r = *.40) in accordance with the results of the overall repeated-measures ANOVA.

Next, Wilcoxon Signed Ranks tests were performed to compare the presence scores at the different vantage points (used as paired variables). Presence was significantly higher at position *A* than at position *B* (*z* = 2.41, *p* = .016, *r* = .35), and was approaching significance when comparing position *A* to position *D* (*z* = 1.73, *p* = .085, *r* = .25). Presence was also higher at positions *B* and *D* than at position *C* (*z* = 3.47, *p* = .001, *r* = .50 and *z* = 3.22, *p* = .001, *r* = .46 respectively). No difference was found between positions *B* and *D* (*z* = 0.76, *p* = .45, *r* = .11). Hence, these results support H2.1, namely that a larger FOV increases the level of presence.

#### Viewing mode

In order to check whether presence was higher for monocular viewing than for binocular viewing (H3.1), we took the median presence scores of all conditions with the *CoPs* coinciding with the vantage points for monocular and binocular viewing separately. Medians and interquartile ranges (in brackets) of these presence scores were 38.0 (*12.0*) for monocular viewing and 37.5 (*11*) for binocular viewing. A Wilcoxon Signed Ranks test showed a significant difference in presence between these two viewing modes (*z* = 2.01, *p* = .04, *r* = .29), even when we only considered the stimuli for which the *CoP* coincided with the vantage point (i.e., again a more limited data set than what was used for the overall repeated-measures ANOVA). This result confirms H3.1 that presence increases for monocular viewing compared to binocular viewing.

#### Center of projection

Since we found a significant interaction between *CoP* and vantage point, we calculated presence scores for different *CoPs* per vantage point by taking the median scores across the three display settings under binocular viewing. Medians and interquartile ranges (in brackets) of the resulting presence scores for the four vantage points are shown in Table 1. Four Friedman tests (i.e., one per vantage point *A*, *B*, *C* and *D*) were conducted to test whether presence was affected by using different *CoPs* (H1.1). The results showed that the *CoP* had an effect on perceived presence at all four vantage points with (*χ^2^*(2, *N* = 24) = 17.8, *p*<.001, *r = *.45) for *A*, (*χ^2^*(2, *N* = 24) = 14.8, *p* = .001, *r = *.39) for *B*, (*χ^2^*(2, *N* = 24) = 18.8, *p*<.001, *r = *.46) for *C*, and (*χ^2^*(2, *N* = 24) = 14.8, *p* = .001, *r = *.39) for *D* respectively.

**Table 1:** Medians and interquartile ranges of presence scores for different projections at four vantage points.

Vantage point (VP)	*CoP* behind VP	*CoP* at VP	*CoP* in front of VP	VP	*CoP* twice angle of VP	*CoP* of *O*	*CoP* at VP
*A*	32.5(21.3)	38.5(12.0)	43.8(17.3)	*B*	25.5(14.0)	31.5(13.5)	34.0(14.0)
*C*	26.0(14.1)	37.5(12.3)	37.5(12.0)	*D*	27.0(18.0)	33.2(13.0)	38.0(11.9)

A series of Wilcoxon Signed Ranks tests was done to compare the difference in presence between different *CoP* renderings. For the vantage points *A* and *C*, presence was higher when the *CoP* was in front of the vantage point than when the *CoP* was at the vantage point with (*z* = 1.81, *p* = .07, *r* = .26) for *A* and (*z* = 3.09, *p* = .002, *r* = .45) for *C*. Presence was also higher for the correct projection rendering, that is *CoP* at the vantage point, than for a *CoP* behind the vantage point with (*z* = 3.50, *p*<.001, *r* = .51) for *A* and (*z* = 2.81, *p* = .007, *r* = .41) for *C*. These results suggest rejection of H1.1 and showed that participants reported a higher level of presence for virtual environments with their *CoP* in front of the vantage point. For the vantage points *B* and *D*, presence was higher for a *CoP* at the vantage point than for a *CoP* at the screen center with (*z* = 2.87, *p* = .004, *r* = .41) for *B* and (*z* = 3.09, *p* = .002, *r* = .45) for *D*. Presence was also higher for a *CoP* at the screen center than for a *CoP* which was at twice the vantage angle with (*z = *2.96, *p* = .003, *r* = .43) for *B* and (*z* = 2.34, *p* = .019, *r* = .34) for *D*. These results support H1.1 that presence increases when the *CoP* is at the vantage point.

#### Display

Medians and interquartile ranges (in brackets) of presence were 40.8 (*15.9*), 35.5 (*12.8*) and 36.0 (*13.8*) for the projector displaying a life-size virtual classroom, the TV displaying a life-size virtual classroom and the TV displaying a scaled-down virtual classroom, respectively. Note that these presence scores were median scores of the four vantage points with the *CoP* matching the vantage point and viewed binocularly. Three Wilcoxon Signed Ranks tests were conducted for comparing the presence scores between the three display settings (used as paired variables). The results showed that the presence experienced in the projector condition was higher than on the TV, independent on whether the TV displayed a life-size virtual classroom (*z = *1.95, *p* = .05, *r* = .28) or a scaled-down virtual classroom (*z* = 2.44, *p* = .015, *r* = .35). There was no significant difference between the TV displaying a life-size virtual classroom and the TV displaying a scaled-down virtual classroom (*z* = 0.36, *p* = .72, *r* = .05). These results support the hypotheses that the projector displaying a life-size virtual environment evoked higher levels of presence than the TV displaying either a life-size (H4.2) or scaled-down virtual environment (H4.1.1). In addition, also hypothesis H4.3, stating that a TV displaying life-size content results in a similar level of presence as a TV displaying scaled-down content, is supported.

### Summary and discussion

Most hypotheses about the effect of FOV, viewing mode and display setting on presence are confirmed. When the vantage point is at *CoP*, presence increases when the participants view the virtual world closer to the screen (H2.1). A projector displaying a life-size virtual classroom evokes a higher level of presence than a TV displaying a life-size (H4.2) or a scaled-down virtual classroom (H4.1.1). No difference in presence is found between the TV displaying a life-size scene and a scaled-down scene (H4.3). It is likely that cue conflicts between perspective and stereovision on a 2D display are stronger and that cues which signal a flat screen (e.g., stereovision, accommodation, etc.) are more apparent when the participants watch the TV screen instead of the projector. Although in the current study, the visual angle per pixel is much smaller on the TV than on the projector, perceived presence is still higher on the projector than on the TV. Therefore, we may conclude that not spatial resolution, but rather the difference in display size dominates the difference in presence between the two display systems. Monocular viewing results in a higher level of presence, which is consistent with the hypothesis that cue conflicts between the perspective cue and stereovision on a 2D display are reduced in the case of monocular viewing (H3.1). For vantage points away from the central axis of the screen, shifting the *CoP* laterally reduces the level of presence which supports H1.1. However, for vantage points along the central axis of the screen, moving the *CoP* to the front of the vantage point improves presence, which rejects H1.1. This finding, therefore, supports the rejection of hypothesis H1.1 in general, namely that presence increases when *CoP* is closer to the vantage point.

The effect on presence of moving the *CoP* away from the vantage point suggests that it matters for the experienced presence how the virtual environment is presented. It is possible that participants have a preference for images with some form of distortion over the veridical one when reporting presence. We hypothesize that the preferred *CoP* is the same as a person's vantage point (H1.3). To test this hypothesis, participants were asked to set their preferred *CoP* for each of the four vantage points.

## Experiment 1: preferred *CoP*

In order to find the participants' preference for a *CoP* at different vantage points and different display settings, the participants were asked to set their own preferred *CoP* at the end of each session in Experiment 1. A wireless keyboard was given to them, and they could press the arrows to control the *CoP* of the virtual scene; that is the left and right arrows were used to control lateral movements, and the up and down arrows were used to control orthogonal movements. The order of the four vantage points and the two viewing modes in each session was random, while the three sessions were counterbalanced over the participants.

### Results

The four vantage points for the TV scaled-down condition were the same as for the TV life-size condition, while the displayed content was the same as used for the projector life-size condition. For the following data analyses, the results of the preferred *CoPs* and the vantage points in TV scaled-down condition were scaled to the corresponding *CoPs* and vantage points as for the projector life-size condition by multiplying with the ratio 4.46. The vantage points are expressed in meters, and so, are numerical values, but since they deviated from normality, non-parametric analyses including Wilcoxon Signed Ranks tests and One-sample Wilcoxon Signed Rank tests were conducted in this section.

To test the effect of viewing mode on preferred *CoP*, we first calculated the deviation between the location of the vantage point and the location of their preferred *CoP* averaged across all the four vantage points and the three display settings. These deviations were calculated as distances along the width dimension (*x*) and the depth dimension (*y*) of the virtual space separately. Medians *M* and interquartile ranges (in brackets) along the width dimension (*x*) and depth dimension (*y*) were *Mx* = −0.02 (0.13), *My = *−0.23 (1.24) and *Mx* = 0.011 (0.11), *My* = −0.50 (1.71) for monocular and binocular viewing respectively. Two Wilcoxon Signed Ranks tests were conducted using deviation distances of both viewing modes as paired variables. No significant difference in perspective preference was found between the two viewing modes along the width dimension (*x*) (*z* = 1.64, *p* = .10, *r* = .24). For the depth dimension (*y*), a significant difference in perspective preference was found (*z* = 3.37, *p* = .001, *r* = .49). During binocular viewing, participants perceived less depth and tended to set the *CoP* closer to the screen, which supports hypothesis H3.2 that monocular viewing (on a 2D display) results in a larger depth impression.

**Figure 6:**
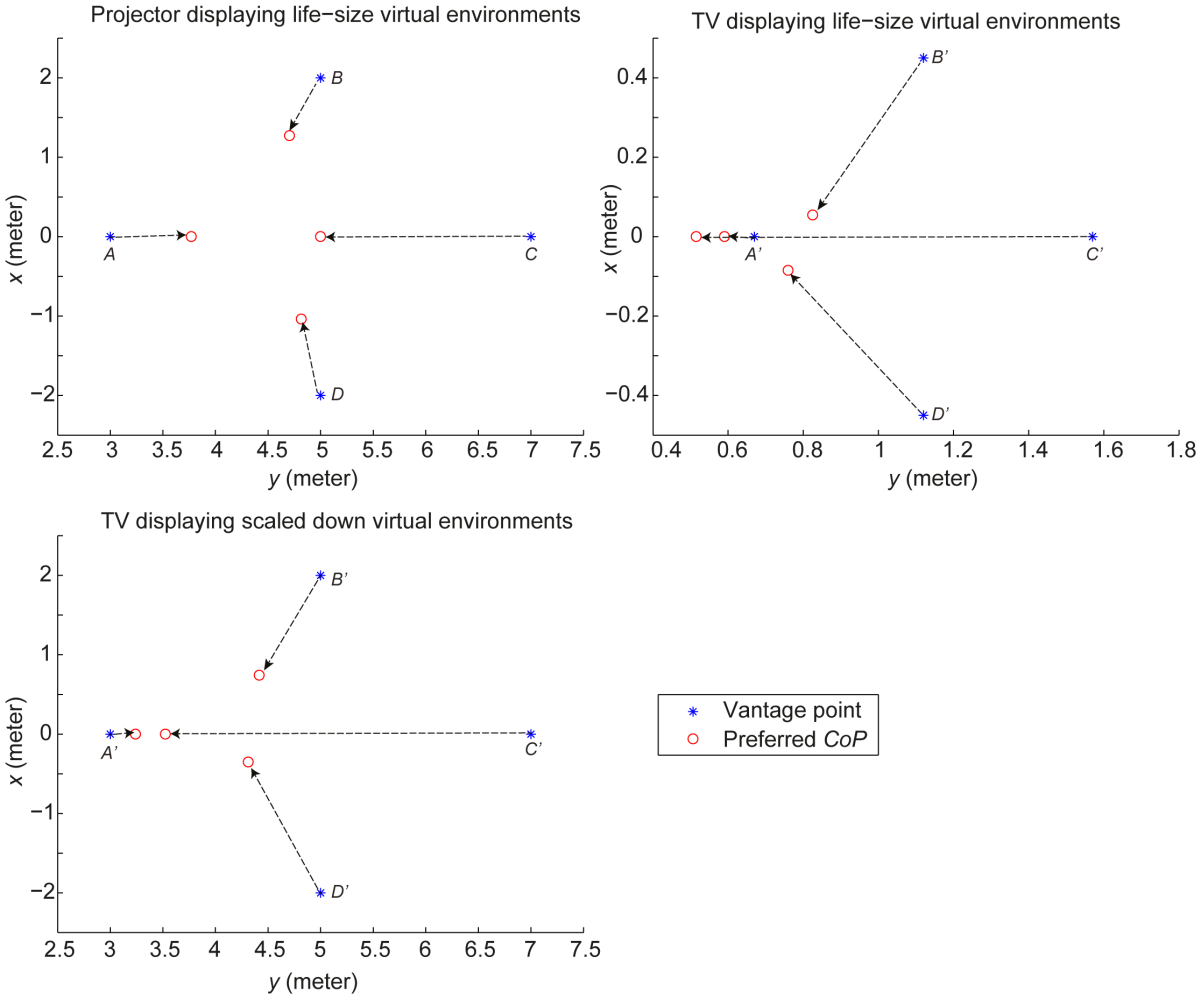
Displacement between the vantage point and the preferred *CoP* at four vantage points across three display settings (Medians).

Next, the deviation distances of the preferred *CoPs* were calculated using binocular data only. Their Medians and interquartile ranges for each vantage point and each display setting are shown in Table 2. The displacement of the preferred *CoP* with respect to the corresponding vantage point is also shown in Figure 6 for the three display settings separately. On these data, we performed a series of One-sample Wilcoxon Signed Rank tests comparing the deviation distances at each vantage point with the test value 0, i.e., to examine whether participants had a preferred *CoP* different from their vantage point (H1.3). The results of the One-sample Wilcoxon Signed Rank tests, given in Table 2, reject H1.3 that participants' preferred *CoPs* are the same as people's vantage points. No significant deviation was found for the width dimension *x* at *A* and *C*. But participants preferred the *CoP* between screen center and their vantage points at *B* and *D*. For the depth dimension *y*, participants chose the *CoP* in front of their vantage point when they sat at *C*. Significant deviations in *y* were also found for *B* in the projector mode and in the TV life-size condition. No significant deviation in *y* was found for *A* and *D*. Also no significant deviation was found for *B* in *y* in the TV scaled-down condition.

**Table 2:** Medians and interquartile ranges of deviation distances in the width dimension (*x*) and depth dimension (*y*) between the preferred *CoP* and the four vantage points across the three display settings in case of binocular viewing; the table also includes the corresponding results of the One-sample Wilcoxon Signed Rank tests with test value 0.

Display settings		*Ax*	*Bx*	*Cx*	*Dx*	*Ay*	*By*	*Cy*	*Dy*
Projector life-size	*Mdn*	0.00	−0.73	0.00	0.96	0.77	−0.30	−2.00	−0.19
	*IQR*	0.02	1.65	0.02	1.71	2.61	1.58	3.29	1.16
	*z*	0.38	−4.08^**^	1.89	4.06^**^	1.89	−2.15^*^	−4.12^**^	−1.40
	*r*	0.05	−0.59	0.27	0.59	0.27	−0.31	−0.59	−0.20
TV life-size	*Mdn*	0.00	−0.40	0.00	0.03	−0.08	−0.30	−1.06	−0.36
	*IQR*	0.01	0.31	0.01	0.29	0.72	0.64	0.52	0.57
	*z*	−1.03	−4.29^**^	0.97	4.10^**^	−0.5	−2.71^*^	−3.06^*^	−2.22
	*r*	−0.15	−0.62	0.14	0.59	−0.07	−0.39	−0.44	−0.32
TV scaling down	*Mdn*	0.00	−1.26	0.00	1.65	0.24	−0.59	−3.47	−0.69
	*IQR*	0.20	1.26	0.04	1.46	2.78	2.41	3.35	2.76
	*z*	1.64	−4.20^**^	1.14	4.16^**^	0.80	−1.80	−3.51^**^	−1.70
	*r*	0.24	−0.61	0.16	0.60	0.12	−0.26	−0.51	−0.25

*H0*: *median* = 0, ^*^ *p<*.05, ^**^ *p*<.001, *n* = 24.

### Summary and discussion

With monocular viewing, participants perceived more depth and preferred the *CoP* further away from the screen than with binocular viewing (which confirms hypothesis H3.2). Hypothesis H1.3, however, is rejected as participants set their preferred *CoP* systematically away from their vantage point. When participants sat away from the screen center (i.e., at vantage points *B* and *D*), they preferred the *CoP* to be between their vantage point and the screen center (i.e., at point *O*). When the participants sat further away in front of the screen center (i.e., at vantage point *C*), they preferred the *CoP* in front of their vantage point.

Participants commented that they tended to set their preferred *CoP* such as to make the room look like an extended part of the experimental room. Examples of typical comments were: "I tried to find the angle that gave the most natural looking 3D view of the room" and "I tried to make it look like the virtual room is an extension of this room as much as possible". The results of the preferred *CoP* suggest that people may have some underestimation or overestimation of the displayed virtual classroom. They also might have ignored "veridicality" of the virtual classroom when they reported their experienced level of presence in Experiment 1. Experiment 2, in which the participants were asked to draw the floor plan of their perceived layout of the virtual classroom, was therefore conducted.

## Experiment 2: space perception

Experiment 2 was conducted to test the effect of *CoP* (H1.2), FOV (H2.2), viewing mode (H3.2) and display (H4.1.2) on perceived shape of the displayed virtual classroom.

### Method

#### Ethics statement

The experiment was approved by the Delft University of Technology Human Research Ethics Committee. Written informed consent was obtained from all participants. Each participant received a small gift for their contribution.

#### Participants

Twenty students, 7 females and 13 males, from the Delft University of Technology participated in the experiment. Their age ranged from 24 to 33 years with a mean of 26.9 (*SD* = 2.13) years. All participants were naive with respect to the hypotheses of experiment 2 and were not involved in the design of the experiment. Half of the participants had already taken part in the first experiment. Fifteen of the participants had experience with virtual reality at least once before they took part in the experiment. Participants viewed the virtual classroom with normal or corrected to normal visual acuity. Means and standard deviations of the participants' visual acuity (Snellen fraction) were 1.16 (0.36), 0.94 (0.38) and 1.28 (0.36) for left eye, right eye and both eyes, respectively.

#### Measurement

**Figure 7:**
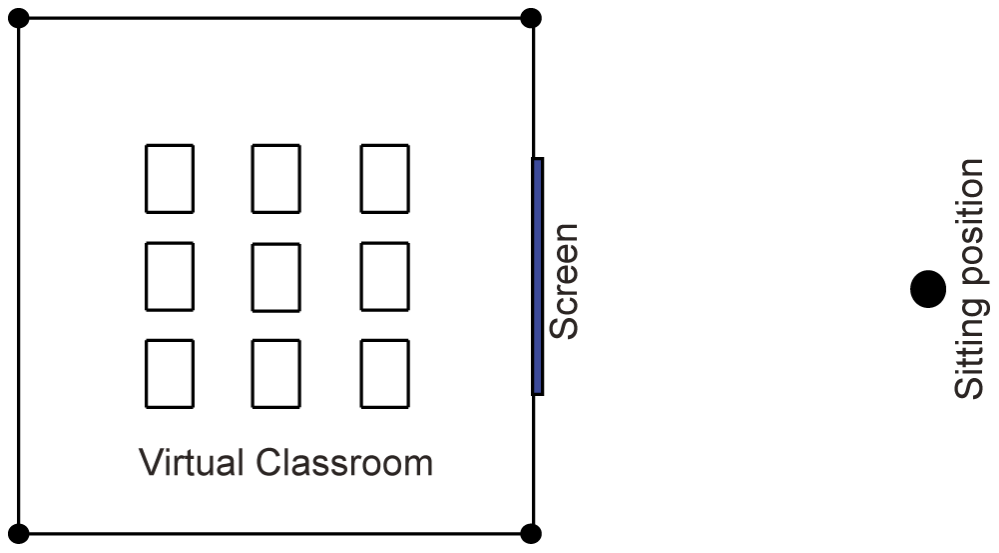
Drawing task interface.

Participants were asked to draw a floor plan of the virtual classroom on a laptop computer. They were also asked to indicate their vantage point in the experimental room. The interface of the drawing task is shown in Figure 7. Only the size and the position of the display screen were fixed in the interface. The initial state of the four corners of the room and the sitting position were default values (8 m in width and 8m in depth for the life-size scene, and 1.7 in width and 1.79 m in depth for the scaled-down scene). As mentioned already in experiment 1, the actual intended size of the life-size scene was 6.6 m in width and 8 m in depth, and the intended size of the scaled-down scene was 1.48 m in width and 1.79 m in depth. The participants' task in this experiment was to draw what they perceived. The participants needed to set the four corners of the room relative to the screen size and set their sitting position (i.e., vantage point). The four corners could be moved independently of each other with the computer mouse. The participant pressed the save button when being satisfied, and the coordinates (*x*,*y*) of the four corners and the sitting position were automatically saved. We also designed the nine tables as a visual aid for drawing the perceived virtual classroom, and the participants could set the corners of the nine tables, but they did not have to. After saving the result of each stimulus, the interface was reset to the default values.

#### Apparatus and procedure

The equipment of Experiment 2 was identical to that of the first experiment. The procedure was virtually the same as in the first experiment except that there were only two sessions. Only the projector displaying a life-size virtual environment and the TV displaying a scaled-down virtual environment were tested; the TV displaying a life-size environment was omitted since it only showed part of the virtual classroom, and so, was not suitable for the drawing task.

#### Analysis

The participants' drawing results were represented as perspective transformation data based on the formula *X = HX’*, where *X* is a vector representing the coordinates of the perceived classroom (i.e., the drawing result), *X’* is the vector representing the coordinates of the intended classroom, and *H* is the transformation matrix [38] (see also Text S2). This equation can be written in homogeneous coordinates as:



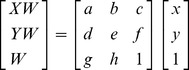
(1)


and 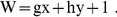
(2)


**Figure 8:**
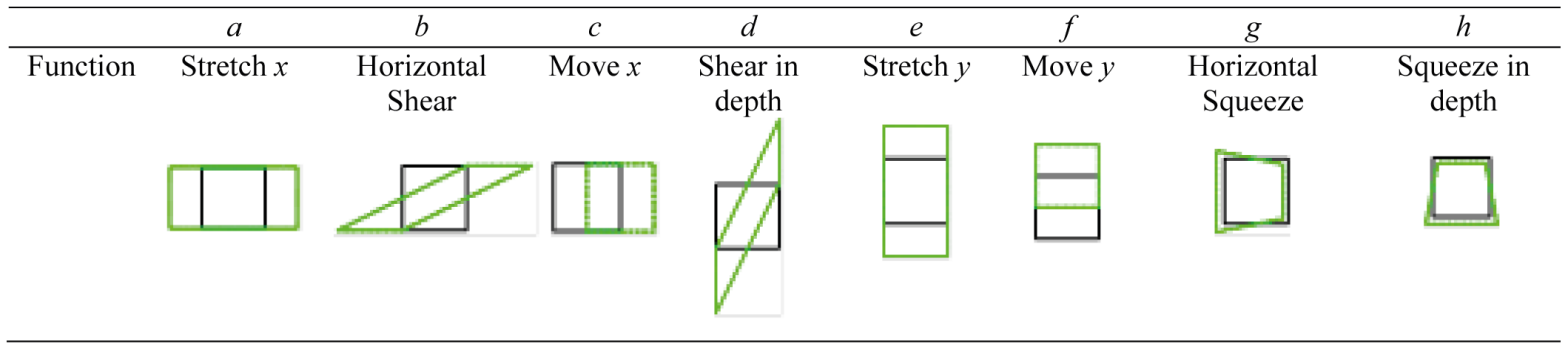
Function of the transformation coefficients in a perspective transformation; the black line is the square in the image plane and the green dashed line is the transformation result.

The effect of each transformation coefficient on a quadrilateral is shown in Figure 8. When *a* and *e* are equal to 1, and the value of the other coefficients is 0, the drawing result is the ideal intended shape.

### Results

The transformation coefficients were calculated based on the formula above for each of the 48 stimuli, resulting from the four vantage points times the three *CoPs* times the two viewing modes times the two display settings. Some of the transformation coefficients deviated from normality, and so non-parametric analyses were conducted, including Wilcoxon Signed Ranks tests for paired comparisons, One-sample Wilcoxon Signed Rank tests for comparing with test values, Friedman tests for comparing several related groups, and factorial repeated-measures ANOVAs on aligned rank data for non-parametric factorial analyses [36].

**Figure 9:**
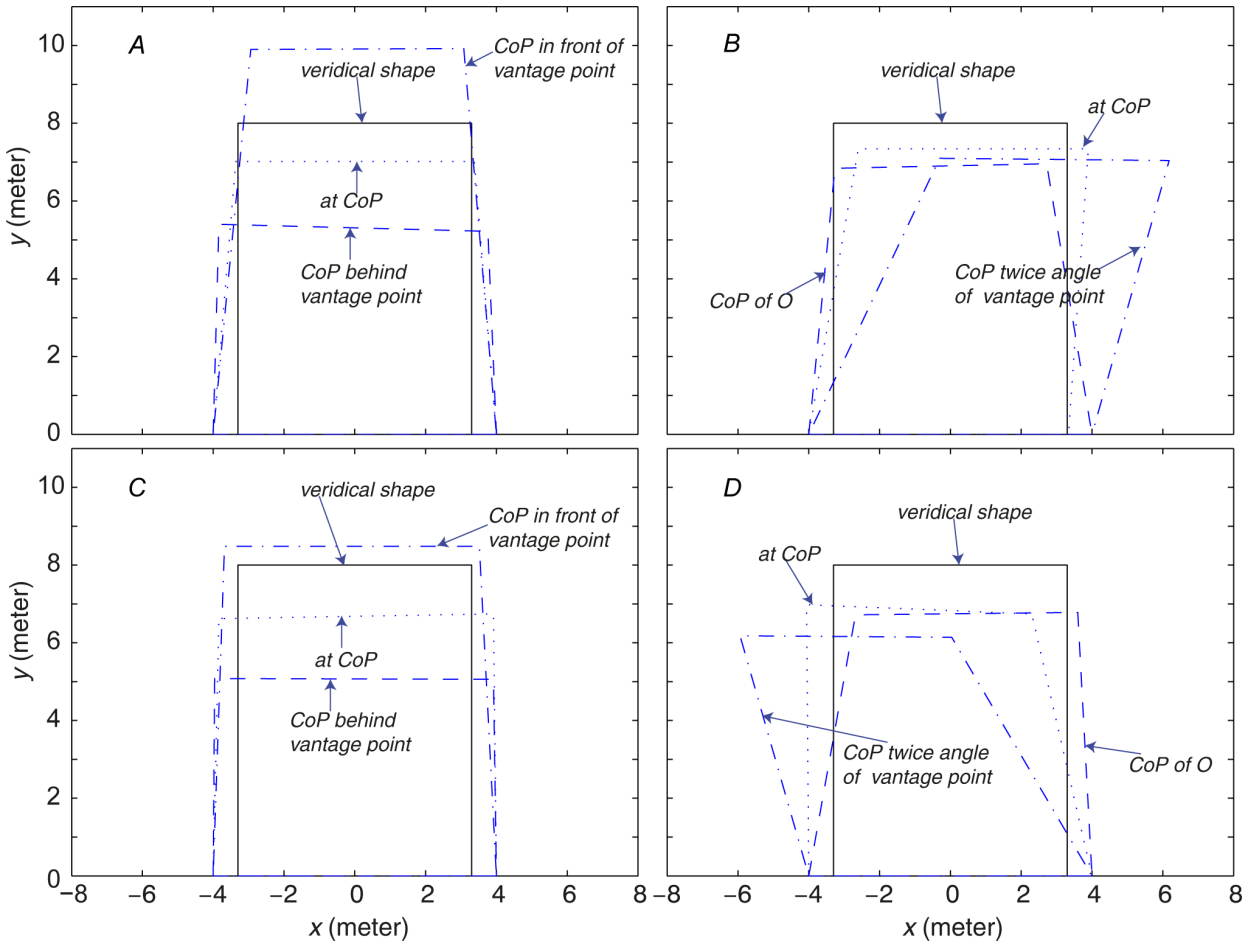
The 12 classrooms (at four vantage points and three *CoPs*) as perceived with binocular viewing on the projector displaying life-size content.

For all stimuli, the coefficients *a*, *e* and *h* were positive, while the coefficients *b*, *c* and *g* had both positive and negative values. One-sample Wilcoxon Signed Rank tests with test value 0 showed no significant difference from 0 for the coefficients *d* (*z* = 0.58, *p* = .56, *r* = .09) and *f* (*z* = 0.29, *p* = .77, *r = *.05), and significant difference from 0 for *h* (*z* = 6.03, *p*<.001, *r = *.87), and the absolute value of the coefficients *b* (*z* = 6.03, *p*<.001, *r = *.87), *c* (*z* = 6.03, *p*<.001, *r = *.87) and *g* (*z* = 6.03, *p*<.001, *r = *.87). One-sample Wilcoxon Signed Rank tests with test value 1 showed significant difference from 1 for the coefficients *a* (*z* = 6.03, *p*<.001, *r = *.87), but no significant difference for *e* (*z* = 1.04, *p* = .30, *r = *.15). Since the interquartile ranges of the coefficients *d* and *f* were zero (i.e., below 1E-15), we excluded *d* and *f* in the rest of the data analyses. In addition, to make all stimuli comparable, we took the absolute value of the coefficients *b*, *c* and *g* to represent the perceived layout of the virtual classroom. To facilitate understanding of the perceived classroom, the drawing results using median coefficients for all 12 stimuli, obtained with binocular viewing of the projector displaying a life-size virtual classroom, are shown in Figure 9.

Six repeated-measures ANOVAs on the aligned ranks data were first conducted using vantage point, viewing mode, *CoP* and display setting as independent factors, and the transformation coefficients *a* (stretch *x*), *b* (horizontal shear), *c* (move *x*), *e* (stretch *y*), *g* (horizontal squeeze) and *h* (squeeze in depth) as dependent variables to find the main effects and their interactions. The results are summarized in Table S2. They show that display setting, viewing mode, *CoP* and vantage point all affected *a*, *b*, *c*, *e*, *g* and *h* significantly; the exceptions were no significant effect of vantage point on *a* and no significant effect of display setting on *b*.

The following subsections give the results of detailed analyses for the hypotheses on the effect of *CoP* (H1.2), FOV by manipulating vantage point (H2.2), viewing mode (H3.2), and display setting (H4.1.2) on space perception.

#### Vantage point

The transformation coefficients for the four vantage points, given in Table 3, were median scores (with the interquartile ranges between brackets) of the two display settings for the condition of binocular viewing with the *CoP* at the vantage point. A series of Friedman tests using vantage point as independent variable and the transformation coefficients as dependent variables were used to test the level of perceived distortion at different vantage points (H2.2). The results showed that (also for this more limited data set) the vantage point had a significant effect on the perceived shape of the virtual classroom with *χ^2^*(3, *N* = 20) = 11.40, *p* = .01, *r = *.29 for the coefficients *a* (stretch *x*); *χ^2^*(3, *N* = 20) = 32.52, *p*<.001, *r = *.67 for *b* (horizontal shear); *χ^2^*(3, *N* = 20) = 27.39, *p*<.001, *r = *.67 for *c* (move *x*); *χ^2^*(3, *N* = 20) = 8.85, *p* = .03, *r = *.24 for *e* (stretch *y*) and *χ^2^*(3, *N* = 20) = 8.36, *p* = .04, *r = *.23 for *g* (horizontal squeeze) respectively.

**Table 3:** Medians and interquartile ranges (in brackets) of the transformation coefficients for stimuli at the four vantage points only using data with the correct perspective (i.e., *CoP* at vantage point) under binocular viewing, but averaged over the two display settings.

Vantage point	*a*	*b*	*c*	*e*	*g*	*h*
	*Stretch x*	*Horizontal shear*	*Move x*	*Stretch y*	*Horizontal squeeze*	*Squeeze in depth*
*A*	1.28 (0.11)	0.008 (0.014)	0.012 (0.026)	1.04 (0.33)	0.002 (0.004)	0.012 (0.079)
*B*	1.17 (0.19)	0.14 (0.14)	0.064 (0.29)	0.96 (0.25)	0.006 (0.010)	0.016 (0.068)
*C*	1.28 (0.26)	0.006 (0.007)	0.016 (0.046)	0.94 (0.39)	0.004 (0.008)	0.009 (0.057)
*D*	1.17 (0.25)	0.15 (0.18)	0.068 (0.22)	1.03 (0.39)	0.006 (0.015)	0.019 (0.11)

To test the effect of vantage point on perceived depth, Wilcoxon Signed Ranks tests were performed on coefficient *e*. Participants perceived more depth at vantage point *A* than at vantage point *C* (*z* = 2.09, *p* = .036, *r* = .33), *B* (*z* = 1.83, *p* = .067, *r* = .29) and *D* (*z* = 1.76, *p* = .079, *r* = .28). No difference was found between the vantage points *B*, *C*, and *D*. These results reject hypothesis H2.2 and suggest that a larger FOV (i.e., the vantage point closer to the screen) induces more depth impression.

#### Viewing mode

We took the median of the transformation coefficients across all correct perspective conditions, i.e., *CoP* at the vantage point, to find representative values for the perceived shape of the classroom under monocular and binocular viewing. Medians and interquartile ranges (in brackets) of these transformation coefficients are shown in Table 4. Wilcoxon Signed Ranks tests were performed with the transformation coefficients in each viewing mode as paired variables. The results showed that there was a difference in perceived depth (i.e., coefficient *e*) of the classroom between these two viewing modes (i.e., *z* = 2.61, *p* = .009, *r* = .38). Participants had a larger depth impression under monocular viewing than under binocular viewing, which supports hypothesis H3.2.

**Table 4:** Medians and interquartile ranges (in brackets) of the transformation coefficients across all stimuli with a correct perspective (i.e., *CoP* at vantage point) obtained for the two viewing modes separately.

Viewing mode	*a*	*b*	*c*	*e*	*g*	*h*
	*Stretch x*	*Horizontal shear*	*Move x*	*Stretch y*	*Horizontal squeeze*	*Squeeze in depth*
Single eye	1.23 (0.12)	0.072 (0.06)	0.062 (0.19)	1.05 (0.22)	0.0055 (0.006)	0.018 (0.07)
Two eyes	1.12 (0.15)	0.074 (0.08)	0.048 (0.16)	1.00 (0.25)	0.0049 (0.008)	0.015 (0.08)

#### Center of projection

The median scores of the two display settings using only the values for binocular viewing were taken as the transformation coefficients for different *CoP* renderings at each of the vantage points. The medians and interquartile ranges for different *CoPs* are shown in Table 5.

**Table 5:** Medians and interquartile ranges (between brackets) of the transformation coefficients for the different *CoPs* at the four vantage points across the two display settings and only for binocular viewing.

Center of projection	*a*	*b*	*c*	*e*	*g*	*h*
	*Stretch x*	*Horizontal shear*	*Move x*	*Stretch y*	*Horizontal squeeze*	*Squeeze in depth*
*C* (*CoP* behind *A*)	1.28 (0.07)	0.009 (0.01)	0.029 (0.040)	0.82 (0.42)	0.005(0.009)	0.009 (0.07)
*A* (*CoP* at *A*)	1.28 (0.11)	0.008 (0.01)	0.012 (0.026)	1.04 (0.33)	0.002 (0.004)	0.012 (0.08)
*E* (*CoP* in front of *A*)	1.27 (0.26)	0.009 (0.02)	0.014 (0.026)	1.65 (1.02)	0.003 (0.003)	0.011 (0.29)
*G* (*CoP* behind *C*)	1.27 (0.19)	0.009 (0.01)	0.016 (0.041)	0.74 (0.35)	0.004 (0.005)	0.008 (0.07)
*C* (*CoP* at *C*)	1.28 (0.26)	0.006 (0.01)	0.016 (0.046)	0.94 (0.39)	0.004 (0.008)	0.009 (0.06)
*A* (*CoP* in front of *C*)	1.25 (0.22)	0.008 (0.01)	0.013 (0.028)	1.17 (0.48)	0.003 (0.003)	0.015 (0.17)
*F* (*CoP* twice angle of *B*)	1.21 (0.20)	0.43 (0.30)	0.14 (0.51)	1.01 (0.22)	0.010 (0.050)	0.035 (0.09)
*O* (*CoP* at *O*)	1.18 (0.26)	0.06 (0.15)	0.057 (0.098)	1.01 (0.33)	0.007 (0.007)	0.030 (0.07)
*B* (*CoP* at *B*)	1.17 (0.19)	0.14 (0.14)	0.064 (0.29)	0.96 (0.25)	0.006 (0.010)	0.016 (0.07)
*H* (*CoP* twice angle of *D*)	1.22 (0.27)	0.40 (0.25)	0.30 (0. 48)	0.97 (0.44)	0.006 (0.045)	0.020 (0.11)
*O* (*CoP* at *O*)	1.28 (0.27)	0.07 (0.08)	0.071 (0.19)	1.07 (0.30)	0.004 (0.015)	0.021 (0.11)
*D* (*CoP* at *D*)	1.17 (0.25)	0.15 (0.18)	0.068 (0.22)	1.03 (0.39)	0.006 (0.015)	0.019 (0.11)

To test whether moving the *CoP* away from the vantage point affected the perceived layout of the virtual classroom (H1.2), we conducted six Friedman tests for each vantage point using the *CoP* as independent variable and the transformation coefficients as dependent variables. For vantage point *A*, the result showed that there was a difference in the coefficients *a* (*χ^2^*(2, *N* = 20) = 14.04, *p* = .001, *r = *.42), *b* (*χ^2^*(2, *N* = 20) = 9.15, *p* = .01, *r = *.33), *c* (*χ^2^*(2, *N* = 20) = 9.36, *p* = .009, *r = *.34), *e* (*χ^2^*(2, *N* = 20) = 34.11, *p*<.001, *r = *.77), *g* (*χ^2^*(2, *N* = 20) = 21.79, *p*<.001, *r = *.55) and *h* (*χ^2^*(2, *N* = 20) = 6.91, *p* = .03, *r = *.28). For vantage point *C*, the Friedman tests showed that there was a significant difference for the coefficient *e* (*χ^2^*(2, *N* = 20) = 38.0, *p*<.001, *r = *.77). For vantage point *B*, the Friedman tests showed that there was a significant difference in the coefficients *a* (*χ^2^*(2, *N* = 20) = 7.21, *p* = .03, *r = *.28), *b* (*χ^2^*(2, *N* = 20) = 25.9, *p*<.001, *r = *.61), and *c* (*χ^2^*(2, *N* = 20) = 14.70, *p* = .001, *r = *.42). Finally, for vantage point *D*, the Friedman tests showed that there was a significant difference in the coefficients *b* (*χ^2^*(2, *N* = 20) = 30.0, *p*<.001, *r = *.77), *c* (*χ^2^*(2, *N* = 20) = 15.1, *p* = .001, *r = *.42), and *e* (*χ^2^*(2, *N* = 20) = 7.90, *p* = .02, *r = *.30).

Next, we used One-sample Wilcoxon signed rank tests to compare the depth impression (i.e., coefficient *e*) to a test value of 1. At vantage point *A*, participants perceived elongated depth when the *CoP* was in front of the vantage point, *z* = 3.92, *p*<.001, *r* = .57, and compressed depth when the *CoP* was behind the vantage point, *z* = 2.17, *p* = .03, *r* = .31. No significant distortion in depth was found when the *CoP* was at the vantage point. The results at vantage point *C* were similar to the ones at vantage point *A*; the One-sample Wilcoxon signed rank tests showed significant compressed depth when the *CoP* was behind the vantage point, *z* = 3.92, *p*<.001, *r* = .57 and elongated depth when the *CoP* was in front of the vantage point, *z* = 2.80, *p* = .005, *r* = .40. There was no significant distortion for the correct perspective. These results support H1.2 that less perspective distortion is perceived when the *CoP* is closer to the vantage point.

Finally, we performed Wilcoxon Signed Ranks tests to compare the various transformation coefficients between the *CoPs* at vantage points *B* and *D* separately. A significantly higher horizontal shear and horizontal translation were perceived for a *CoP* at twice the angle of the vantage point than for a *CoP* at the vantage point and for a *CoP* at the screen center. These conclusions were observed both at the vantage points *B* and *D* (all *p*<.001 and *r* >.56). We found no significant difference in horizontal shear (i.e., coefficient *b*) between a *CoP* at the vantage point and a *CoP* at the screen center, neither for vantage point *B* nor for vantage point *D*. These results reject hypothesis H1.2, since they show no significant difference between a *CoP* at the vantage point and a *CoP* at the screen center in perceived shear.

#### Display

Here the median transformation coefficients of the four vantage points for each of the two display settings were taken using only the data under a geometrically correct perspective (i.e., *CoP* at vantage point) viewed binocularly. The corresponding medians and interquartile ranges for the projector displaying a life-size virtual classroom and the TV displaying a scaled-down virtual classroom are shown in Table 6. Six Wilcoxon Signed Ranks tests were conducted using the transformation coefficients in each display setting as paired variables; the results are also included in Table 6. Participants perceived more stretched depth (i.e., coefficient *e*) in the TV displaying a scaled-down virtual classroom than in the projector displaying a life-size virtual classroom, which rejects hypothesis H4.1.2.

**Table 6:** Medians and interquartile ranges (between brackets) of the transformation coefficients per display setting across the four vantage points only using the data of the correct perspective (i.e., *CoP* at vantage point) viewed binocularly; the table also includes the results of Wilcoxon Signed Ranks tests (*n = *20).

	*a*	*b*	*c*	*e*	*g*	*h*
	*Stretch x*	*Horizontal shear*	*Move x*	*Stretch y*	*Horizontal squeeze*	*Squeeze in depth*
Projector	1.19	0.084	0.062	0.97	0.002	0.005
	(0.13)	(0.077)	(0.27)	(0.33)	(0.003)	(0.03)
TV	1.25	0.066	0.034	1.00	0.008	0.026
	(0.25)	(0.089)	(0.04)	(0.35)	(0.014)	(0.13)
Wilcoxon Signed Ranks tests	*z = *−2.13	*z = *0.93	*z = *2.88	*z = *−2.20	*z* = −3.92	*z = *−3.92
	*p = *.03	*p = *.35	*p = *.004	*p = *.03	*p<*.001	*p<*.001
	*r = *.31	*r = *.13	*r = *.42	*r = *.32	*r = *.57	*r = *.57

When using transformation coefficients, the size of the virtual classroom was compared always on a relative scale, only giving an indirect estimation of the real physical size of the virtual classroom. The relative dimensions, however, can be translated into physically real dimension for the perceived shape of the virtual classroom by means of the transformation coefficients. Doing so, let us compare the perceived physical depth of the virtual classroom for the projector (*Mdn* = 7.04 m, *IQR* = 2.37 m) to that for the TV (*Mdn* = 1.63 m, *IQR* = 0.51 m) with a Wilcoxon Signed Ranks test. The result shows that participants perceived a significantly larger virtual classroom on the projector displaying a life-size scene than on the TV displaying a scaled-down scene (*z* = 3.92, *p*<.001, *r* = .57). These results indicate that despite the stretched depth on the TV is larger than the projector, the virtual classroom is still perceived as much larger in depth on the projector than on the TV.

### Summary and discussion

In summary, when the *CoP* coincides with the vantage point, participants perceive more depth with monocular viewing compared to binocular viewing, supporting hypothesis H3.2. Hypothesis H1.2, which states that less distortion is perceived when the *CoP* is closer to the vantage point, is not fully supported. It is supported for vantage points in front of the screen center (i.e., vantage points *A* and *C*), where no significant distortion is found for the correct perspective, i.e., a *CoP* at the vantage point, but where less depth is perceived when the *CoP* is behind the vantage point and more depth when the *CoP* is in front of the vantage point. However, for vantage points away from the screen center (i.e., *B* and *D*), participants perceive a less distorted virtual classroom when shifting the *CoP* to the center of the screen (i.e., to point *O*) than when shifting the *CoP* laterally away from the screen center. No significant difference is found between a *CoP* at the vantage point and a *CoP* at the screen center. The latter can explain why participants set their preferred *CoP* between the respective vantage points *B* and *D* and the screen center. Hypothesis H2.2 is rejected since more depth is perceived at vantage point *A* (i.e., with a larger FOV) than at vantage point *C*. The latter can explain why participants set their preferred *CoP* in front of their vantage point when they sit farther away in front of the screen (i.e., at vantage point *C*).

The projector displaying a life-size virtual classroom results in a less stretched depth of the classroom than the TV displaying a scaled-down virtual classroom, which rejects hypothesis H4.1.2. Participants perceive a stretched version of the scaled-down virtual classroom both in width and depth. Although cue conflicts between stereovision and perspective are stronger when the viewer is close to the screen, the shape of the scaled-down virtual classroom is perceived larger than intended, but still it is perceived much smaller than the life-size virtual classroom on the projector.

## Relationship between Presence, Preferred Center of Projection and Perceived Distortion

**Figure 10:**
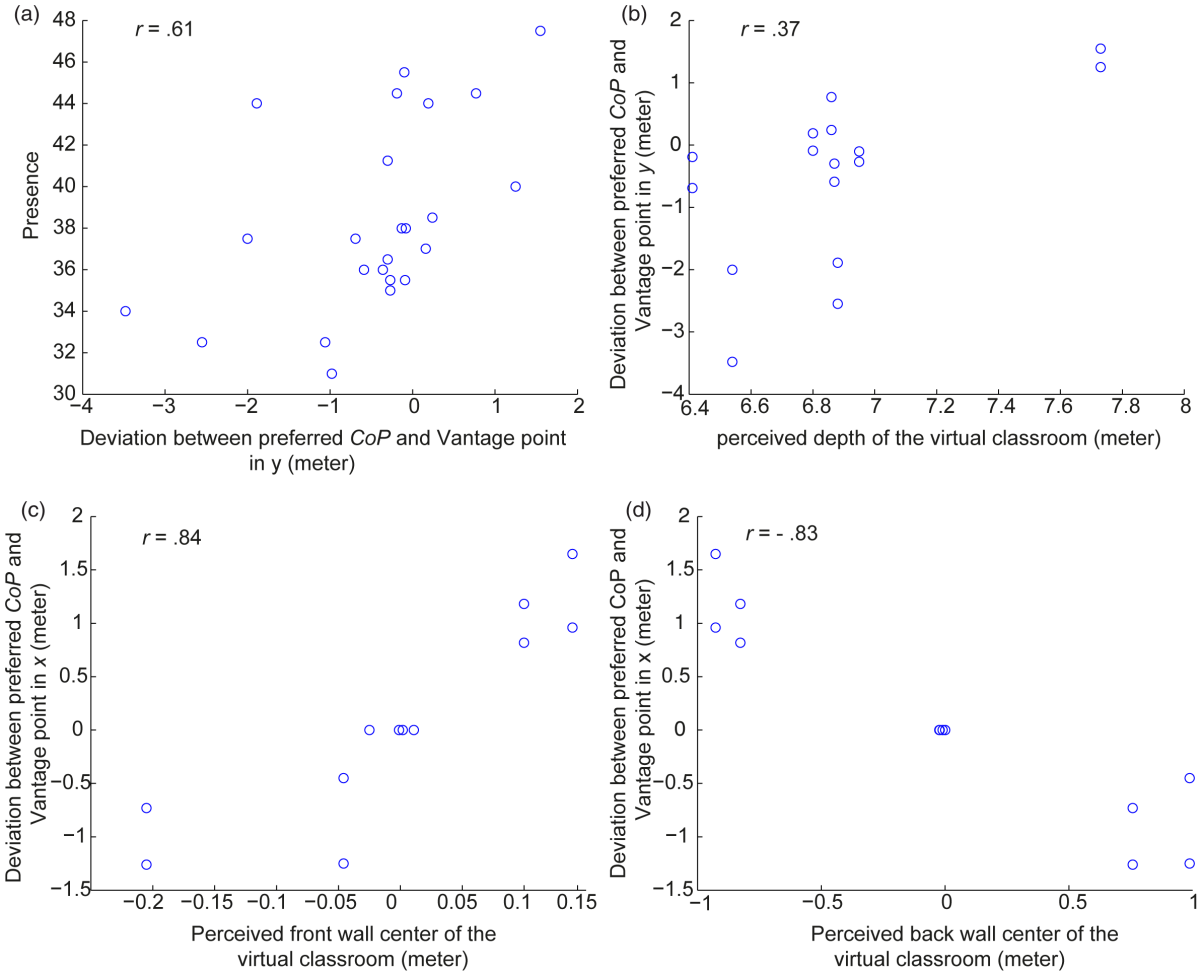
Relationships between presence, preferred *CoP* and perceived distortion (Spearman correlation).

The relationship between presence and preferred *CoP* was tested using Spearman correlation analysis between the median presence rating across participants at the correct perspective (i.e., for the data with the *CoP* at the vantage point) and the median value of the deviation between preferred *CoP* and the vantage point (i.e., subtracting vantage point from preferred *CoP*) across participants. The analysis showed a significant correlation between presence and the deviation distance of the *CoP* in the depth direction (i.e., the *y*-direction) (*r*(24) = .61, *p* = .002) (Figure 10a). No significant correlation between presence and the deviation distance of the *CoP* in the *x*-direction (i.e., the width direction) was found. This result suggests that more presence was reported for the stimulus with a preferred *CoP* behind the vantage point than for the stimulus with a preferred *CoP* in front of the vantage point.

To test the relationship between preferred *CoP* and perceived layout of the virtual classroom, we conducted Spearman correlation analyses between the deviation distances of the preferred *CoP* from the vantage point as obtained in Experiment 1 and the perceived layout of the classroom at the correct perspective (i.e., with *CoP* at the vantage point) as obtained in Experiment 2. Note that the perceived physical sizes of the TV displaying a scaled-down virtual environment were enlarged by the ratio 4.46. No significant correlation between deviation distance of the preferred *CoP* in the depth direction and perceived depth *y* of the virtual classroom was found (*r*(16) = .37, *p* = .16) (Figure 10b). However, significant correlations were found between the deviation distance of the preferred *CoP* in *x*-direction and the perceived front wall center, which was the average of the left and right corners at the front in *x*-direction (*r*(16) = .84, *p*<.001), on the one hand, and the perceived back wall of the virtual classroom, which was the average of the left and right corners at the back in *x*-direction (*r*(16) = −.83, *p*<.001), on the other hand (see Figure 10c-d). Sitting laterally away from the screen center results in perceived lateral shears of the room in the direction opposite to the displacement, and therefore, the participants tend to set their preferred *CoP* closer to the screen center in order to make the classroom look more realistic (Figure 10c-d).

The relationship between presence and perceived shape of the virtual classroom (as addressed in hypothesis H1.4) was tested using regression analysis (Stepwise) between presence scores as obtained in Experiment 1 and the transformation coefficients for each vantage point as obtained in Experiment 2 (see Table 3). Note that the regression analyses were based on the ranked data of all the variables. The results are summarized in Table 7 and show that for both vantage points *A* and *C*, the coefficient *a* (i.e., a stretch in *x*-direction) significantly predicts the level of experienced presence, which means that a perceived stretched width of the virtual classroom explains a reduction in the level of experienced presence. At vantage point *C*, the regression function also contains a significant contribution of the coefficient *e* (i.e., a stretch in *y*-direction), which implies that also a perceived elongated depth is associated with an increase in the level of experienced presence. This latter result is in accordance with the result that participants experienced a higher level of presence and a perceived elongated depth when the *CoP* was in front of the vantage point. For the vantage points away from the screen center (i.e., *B* and *D*), experienced presence is significantly related to coefficient *g* (i.e., a horizontal squeeze). These results confirm hypothesis H1.4, stating that the sense of presence can be predicted from the perceived layout of the virtual environment. Part of the transformation coefficients are negatively correlated with the participants’ feelings of presence, but others such as the perceived elongated depth (i.e., coefficient *e*) are positively related to the experienced presence.

**Table 7:** Regression analysis (Stepwise) predicting the level of experienced presence as a function of the perceived shape of the room for four different vantage points (*n* = 12).

Vantage point	Variable	*B*	*SE B*	*β*	*R* ^2^	*F*
*A*	*a-*stretch *x*	−1.00	0.09	−1.00^**^	.93	56.71
	*b-*horizontal shear	0.35	0.09	0.35^*^		
						
*B*	*g-*horizontal squeeze	−0.75	0.21	−0.75^*^	.56	12.72
						
*C*	*e-*stretch *y*	0.83	0.08	0.83^**^	.95	78.85
	*a-*stretch *x*	−0.50	0.08	−0.50^**^		
						
*D*	*g-*horizontal squeeze	−0.91	0.13	−0.91^**^	.83	47.62

Note: ^*^ *p*<0.01; ^**^ *p*<.001.

## General Discussion

We conducted two experiments to test four hypotheses in this study. To test the difference between monocular and binocular viewing, we used non-stereoscopic displays, and thus, had to consider the depth cue conflict between stereovision and perspective: the perspective showed a three-dimensional world, while the stereovision and accommodation cues signaled a flat image. Because this conflict between stereovision and perspective was removed or minimized in the case of monocular viewing, we found a higher level of experienced presence, a larger depth impression and a preferred *CoP* closer to the vantage point for monocular viewing than for binocular viewing. These results confirm hypothesis H3 and suggest that when the cue conflict between stereovision and perspective is reduced or when a stereoscopic depth cue is applied, a more accurate depth perception and higher levels of experienced presence can be achieved.

At close viewing distances, the cue conflict between stereovision and perspective is stronger [26]. This may be one of the reasons that even with the same field of view and the same content, a larger screen results in a better performance than a small one in spatial navigation tasks [27]. Similarly, in the current experiments, participants experienced a higher level of presence with the projector displaying a life-size virtual classroom than with the TV displaying the same content with the same field of view, and so at a shorter viewing distance. This observation supports hypothesis H4.1.1.

The variation in cue conflict with viewing distance may also explain why people experience a higher level of presence viewing a projector displaying a life-size virtual environment compared to a TV displaying a life-size virtual environment, which supports hypothesis H4.2. With the same field of view, showing a smaller part of the life-size virtual classroom on the TV than on the projector may be another possible reason for a lower level of presence in the TV life-size condition. However, no difference in presence between a TV displaying a life-size scene and a TV displaying a scaled-down scene was found (supports hypothesis H4.3). It seems that without any required task, a TV displaying either a life-size or scaled-down scene does not affect experienced feelings of presence.

When the *CoP* coincided with the vantage point, the participants experienced higher levels of presence - which supports hypothesis H2.1 - and more depth - which rejects hypothesis H2.2 - for a vantage point closer to the display screen. No interaction between viewing mode and vantage point on presence was found. So, even though the cue conflict between stereovision and perspective was stronger when the viewer was closer to the screen, its effect on experienced presence was compensated by the increased FOV at the closer vantage point. In other words, our results suggest that the effect of FOV on presence is more dominant than the effect of cue conflict.

Our results also showed that for vantage points in front of the screen center (i.e., *A* and *C*), shifting the *CoP* orthogonally in front of the vantage point improved the experienced presence, which rejects hypothesis H1.1. For vantage points away from the screen center, (i.e., *B* and *D*) participants experienced the highest level of presence for content rendered with a *CoP* at the vantage point. Shifting the *CoP* to the center of the screen evoked a higher level of presence and a less distorted virtual classroom compared to shifting the *CoP* further away from the screen center beyond the vantage point. These results imply that for groups viewing a display in which the content is rendered with a *CoP* in front of the screen center, people sitting closer to the screen center experience more presence than people sitting away from the screen center.

Although participants adjusted their preferred setting of the *CoP* such as to make the virtual classroom look more like an extension of the experimental room, significant displacements of the preferred *CoP* from their vantage point were found, which rejects hypothesis H1.3. For vantage points away from the screen center, (i.e., *B* and *D*), participants adjusted the *CoP* of the virtual classroom between the vantage point and the screen center. As such, these results reject the theory that displacements between *CoP* and vantage point do not significantly affect the perception of the conveyed virtual space [13], [15], and the geometrical analyses [6], [7]. However, our results are in accordance with the studies reported by Todorovic [11] and Pont et al. [10], which state that the effect of displacement between vantage point and *CoP* is smaller than what the geometrical analyses predict.

We found a significant relationship between presence and some transformation coefficients, as such supporting hypothesis H1.4 that the sense of presence can be predicted by the perceived layout of the virtual environment. In general, perceived distortions in the perspective are associated with a reduction in experienced presence. For vantage points in front of the screen center, a more stretched width predicts a lower level of presence. For vantage points away from the screen center, a more squeezed shape indicates a lower level of presence. These results suggest that the way the virtual classroom is presented is crucial for the level of experienced presence. Some distortions in perspective, however, such as an elongated depth may improve the sense of presence in virtual environments. This latter result is in line with the result that when the *CoP* was in front of the vantage point, participants perceived elongated depth and experienced a higher level of presence.

It appears that a wider view on the virtual environment (i.e., with the *CoP* in front of the vantage point) is an important aid for many spatial tasks, helping especially with cognitive map construction when the visual complexity of a display or the demands of a task increase [39]. Steinicke et al. [40] suggested that designers may consider modifying the displayed virtual reality scene dynamically by manipulating the displacement between vantage point and *CoP* depending on the type of task. For certain applications which mainly focus on aspects like aesthetics, enjoyment or task performance, such as games or navigation tasks, it may not be required that users perceive the virtual scene in such a way as they would perceive the corresponding real world scene. Rendering some perspective distortions such as elongated depth may improve the experienced presence in these virtual environments. However, if the primary task of a user requires perceiving the virtual scene as its real world counterpart, e.g., during training with a flight simulator, in which case perception of the virtual objects should match those perceived in the real world, the virtual environment should be displayed with the *CoP* at the vantage point.

No specific task such as giving a talk or playing a game was requested during the immersion in our virtual environment. Task is known to be able to have a significant effect on presence [41], [42]. For example, Hoffman et al. [41] found that presence can be significantly improved when a virtual chess is put in a meaningful way for experienced chess players. Ling et al. [42] showed a significant higher level of presence in the virtual public speaking world than in the neutral world, even though both virtual environments were designed and displayed with the same technology. Future work needs to be done to check whether our findings still apply to virtual reality applications involving a high cognitive load.

In conclusion, the results in the current study show that field of view, viewing mode, *CoP* and screen size all significantly affect the sense of presence and the perceived layout of the virtual environment. Presence and the preferred *CoP* are both linearly related to the perceived perspective distortion of the virtual environment. Our results suggest that the way in which virtual worlds are presented affects people's perception of the virtual environment and that this is critical for the level of experienced presence. More depth is perceived and a higher level of presence is experienced in virtual environments that appear more elongated than intended. This conclusion provides a way to improve the sense of presence in virtual environments on especially small screen displays; i.e., by showing elongated environments. Our results also show the possibility to improve applications such as games and virtual reality exposure therapy at home.

## Supporting Information

Table S1 **Hypotheses in experiments. (PDF)**
(PDF)

Table S2 **Results of repeated-measures ANOVAs on the aligned ranks data using vantage point, viewing mode, *CoP* and display setting as independent factors, and the transformation coefficients as dependent variables. (PDF)**
(PDF)

Text S1 **Selected items of the presence questionnaire. (PDF)**
(PDF)

Text S2 **Perspective transformation. (PDF)**
(PDF)
