# Supplementary material for: The Effect of Perspective on Presence and Space Perception
Source: PLoS One. 2013 Nov 6;8(11):e78513. doi: 10.1371/journal.pone.0078513 (PMC3819378; doi:10.1371/journal.pone.0078513)
Supplement: Table S1 — Hypotheses in experiments. (PDF) (PDF) [file pone.0078513.s001.pdf]

The effect of perspective on presence and space perception

Yun Ling, Harold T. Nefs, Willem-Paul Brinkman, Chao Qu, Ingrid Heynderickx

Supporting Table S1

Hypotheses in experiments

	**Experiment one** (presence and preferred *CoP*)	**Experiment two** (Perceived space layout of the virtual classroom)
H1-*CoP*	The level of presence increases (H1.1) when the *CoP* moves closer to the vantage point.<br><br>A person's preferred *CoP* equals his/her vantage point (H1.3).	Perceived distortion decreases (H1.2) when the *CoP* moves closer to the vantage point.
	The sense of presence can be predicted from the perceived layout of the virtual environment (H1.4).	
H2-FOV	When the *CoP* is at the vantage point, a larger FOV increases the level of presence (H2.1).	When the *CoP* is at the vantage point, a larger FOV has no effect on the perceived shape of the virtual classroom (H2.2).
H3-Viewing mode	Monocular viewing results in a higher level of presence (H3.1) than binocular viewing on a 2D display.	Monocular viewing results in more depth impression (H3.2) than binocular viewing on a 2D display.
H4-Display settings	When the *CoP* is at the vantage point, individuals report higher levels of presence on the projector displaying a life-size virtual world than on the TV displaying the same content with the same FOV (H4.1.1).<br><br>When the *CoP* is at the vantage point, individuals report higher levels of presence in a life-size virtual world when exposed to a projector than when exposed to a TV displaying a life-size virtual world (H4.2) and experience similar levels of presence on the TV when it displays a life-size virtual world or a scaled-down virtual world with the same FOV (H4.3).	When the *CoP* is at the vantage point, individuals report a more stretched depth on the projector displaying a life-size virtual world than on the TV displaying the same content with the same FOV (H4.1.2).
