# Supplementary material for: The Effect of Perspective on Presence and Space Perception
Source: PLoS One. 2013 Nov 6;8(11):e78513. doi: 10.1371/journal.pone.0078513 (PMC3819378; doi:10.1371/journal.pone.0078513)
Supplement: Table S2 — Results of repeated-measures ANOVAs on the aligned ranks data using vantage point, viewing mode, CoP and display setting as independent factors, and the transformation coefficients as dependent variables. (PDF) (PDF) [file pone.0078513.s002.pdf]

The effect of perspective on presence and space perception

Yun Ling, Harold T. Nefs, Willem-Paul Brinkman, Chao Qu, Ingrid Heynderickx

Supporting Table S2

Results of repeated-measures ANOVAs on the aligned ranks data using vantage point, viewing mode, *CoP* and display setting as independent factors, and the transformation coefficients as dependent variables

Table S2.1. Results of repeated-measures ANOVAs on the aligned ranks data: the main and interaction effects of the independent factors (i.e., Display, Viewing mode, *CoP* and Vantage point), on *a* (stretched width) and *b* (horizontal shear) of the perceived shape of the virtual room.

		a (stretched width)			*b* (horizontal shear)		
		F	*p*	η^2	*F*	*p*	η^2
Display	$F(1,19)$	19.19	<.001	.50	4.14	.06	.18
Viewing mode	$F(1,19)$	4.50	.05	.19	29.58	<.001	.61
CoP	$F(2,38)$	5.14	.02	.21	68.74	<.001	.78
Vantage point	$F(3,57)$	1.35	.27	.07	121.85	<.001	.87
Display * Viewing mode	$F(1,19)$	2.76	.11	.13	0.91	.35	.05
Display * *CoP*	$F(2,38)$	0.89	.42	.05	7.20	.002	.28
Viewing mode * *CoP*	$F(2,38)$	0.63	.54	.03	5.84	.01	.24
Display * Viewing mode * *CoP*	$F(2,38)$	0.28	.76	.02	11.20	<.001	.37
Display * Vantage point	$F(3,57)$	1.97	.13	.09	5.47	.01	.22
Viewing mode * Vantage point	$F(3,57)$	0.94	.43	.05	8.30	.001	.30
Display * Viewing mode * Vantage point	$F(3,57)$	2.21	.10	.10	0.79	.51	.04
CoP * Vantage point	$F(6,114)$	2.19	.05	.10	61.38	<.001	.76
Display * *CoP* * Vantage point	$F(6,114)$	2.19	.05	.10	2.97	.04	.14
Viewing mode * *CoP* * Vantage point	$F(6,114)$	0.95	.43	.05	7.33	<.001	.28
Display * Viewing mode * *CoP* * Vantage point	$F(6,114)$	0.53	.79	.03	4.90	.01	.21

Table S2.2. Results of repeated-measures ANOVAs on the aligned ranks data: the main and interaction effects of the independent factors (i.e., Display, Viewing mode, *CoP* and Vantage point) on *c* (horizontal move *x*) and *e* (stretch in depth *y*) of the perceived shape of the virtual room.

		c (horizontal move *x*)			*e* (stretch in depth *y*)		
		F	*p*	η^2	*F*	*p*	η^2
Display	*F*(1,19)	269.15	<.001	.93	6.92	.02	.27
Viewing mode	*F*(1,19)	6.96	.02	.27	10.04	.01	.35
CoP	*F*(2,38)	86.59	<.001	.82	92.36	<.001	.83
Vantage point	*F*(3,57)	68.90	<.001	.78	20.08	<.001	.51
Display * Viewing mode	*F*(1,19)	0.04	.85	<.01	0.01	.94	<.01
Display * *CoP*	*F*(2,38)	116.82	<.001	.86	2.87	.07	.13
Viewing mode * *CoP*	*F*(2,38)	6.03	.01	.24	0.60	.55	.03
Display * Viewing mode * *CoP*	*F*(2,38)	1.95	.16	.09	0.13	.88	.01
Display * Vantage point	*F*(3,57)	43.83	<.001	.70	1.08	.37	.05
Viewing mode * Vantage point	*F*(3,57)	2.22	.12	.10	1.44	.24	.07
Display * Viewing mode * Vantage point	*F*(3,57)	2.57	.06	.12	1.72	.17	.08
CoP * Vantage point	*F*(6,114)	40.34	<.001	.68	39.81	<.001	.68
Display * *CoP* * Vantage point	*F*(6,114)	46.47	<.001	.71	0.38	.89	.02
Viewing mode * *CoP* * Vantage point	*F*(6,114)	2.13	.09	.10	1.61	.15	.08
Display * Viewing mode * *CoP* * Vantage point	*F*(6,114)	2.96	.03	.14	1.20	.31	.06

Table S2.3. Results of repeated-measures ANOVAs on the aligned ranks data: the main and interaction effects of the independent factors (i.e., Display, Viewing mode, *CoP* and Vantage point) on *g* (horizontal squeeze) and *h* (squeeze in depth) of the perceived shape of the virtual room.

		g (horizontal squeeze)			*h* (squeeze in depth)		
		F	*p*	η^2	*F*	*p*	η^2
Display	*F*(1,19)	916.81	<.001	.98	109.00	<.001	.85
Viewing mode	*F*(1,19)	8.87	.01	.32	7.36	.01	.28
CoP	*F*(2,38)	69.94	<.001	.79	42.70	<.001	.69
Vantage point	*F*(3,57)	68.94	<.001	.78	3.88	.04	.17
Display * Viewing mode	*F*(1,19)	0.05	.84	<.01	11.70	.00	.38
Display * *CoP*	*F*(2,38)	208.04	<.001	.92	52.13	<.001	.73
Viewing mode * *CoP*	*F*(2,38)	26.68	<.001	.58	21.81	<.001	.53
Display * Viewing mode * *CoP*	*F*(2,38)	7.35	.002	.28	22.60	<.001	.54
Display * Vantage point	*F*(3,57)	80.82	<.001	.81	5.35	.01	.22
Viewing mode * Vantage point	*F*(3,57)	2.16	.10	.10	12.44	<.001	.40
Display * Viewing mode * Vantage point	*F*(3,57)	3.71	.02	.16	12.67	<.001	.40
CoP * Vantage point	*F*(6,114)	56.91	<.001	.75	37.66	<.001	.67
Display * *CoP* * Vantage point	*F*(6,114)	107.15	<.001	.85	29.66	<.001	.61
Viewing mode * *CoP* * Vantage point	*F*(6,114)	3.34	.01	.15	9.15	<.001	.33
Display * Viewing mode * *CoP* * Vantage point	*F*(6,114)	0.47	.83	.02	12.64	<.001	.40
