# Supplementary material for: The Effect of Perspective on Presence and Space Perception
Source: PLoS One. 2013 Nov 6;8(11):e78513. doi: 10.1371/journal.pone.0078513 (PMC3819378; doi:10.1371/journal.pone.0078513)
Supplement: Text S1 — Selected items of the presence questionnaire. (PDF) (PDF) [file pone.0078513.s003.pdf]

The effect of perspective on presence and space perception

Yun Ling, Harold T. Nefs, Willem-Paul Brinkman, Chao Qu, Ingrid Heynderickx

Supporting Text S1

Selected items of the presence questionnaire

Note that we can’t disclose the full questionnaire because of copyright issues. Questions that have been modified with respect to the original questionnaire and the added question are:

(1) Watching the virtual classroom gave me a sense of ‘an actual room being there’. 1. Not at all. 7. Very much.

(2) Did you have the impression that the virtual classroom was an extension of this room? 1. Not at all. 7. Very much.

(3) Did you have the impression that you could have walked into the virtual classroom? 1. Not at all. 7. Very much.

(4) Did the virtual classroom appear to be visualized on a screen, or did you have the impression that it was a room next door? 1. Visualized image. 7. A real room next door.
