# Supplementary material for: The Effect of Perspective on Presence and Space Perception
Source: PLoS One. 2013 Nov 6;8(11):e78513. doi: 10.1371/journal.pone.0078513 (PMC3819378; doi:10.1371/journal.pone.0078513)
Supplement: Text S2 — Perspective transformation. (PDF) (PDF) [file pone.0078513.s004.pdf]

The effect of perspective on presence and space perception

Yun Ling, Harold T. Nefs, Willem-Paul Brinkman, Chao Qu, Ingrid Heynderickx

Supporting Text S2

Perspective transformation

The participants' drawing results were represented as perspective transformation data based on the formula $X = HX'$, where X is a vector representing the coordinates of the perceived classroom (i.e., the drawing result), X' is the vector representing the coordinates of the intended classroom, and H is the transformation matrix [1]. This equation can be written in homogeneous coordinates as:

$$\begin{bmatrix} XW \\ YW \\ W \end{bmatrix} = \begin{bmatrix} a & b & c \\ d & e & f \\ g & h & 1 \end{bmatrix} \begin{bmatrix} x \\ y \\ 1 \end{bmatrix},$$

and $W = gx + hy + 1$.

Rewrite this formula as:

$$\begin{bmatrix} X \\ Y \\ 1 \end{bmatrix} = \frac{\begin{bmatrix} a & b & c \\ d & e & f \\ g & h & 1 \end{bmatrix} \begin{bmatrix} x \\ y \\ 1 \end{bmatrix}}{\begin{bmatrix} g & h & 1 \end{bmatrix} \begin{bmatrix} x \\ y \\ 1 \end{bmatrix}}$$

By multiplying each side of the equation by the denominator we get

$$X(gx + hy + 1) = ax + by + c \,,$$

and

$$Y(gx + hy + 1) = dx + ey + f.$$

Then multiplying through by X and Y gives us:

$$gXx + hXy + X = ax + by + c \,,$$

and

$$gYx + hYy + Y = dx + ey + f.$$

By isolating the naked X and Y terms on the left and we get:

$$X = ax + by + c - gXx - hXy,$$

and

$$Y = dx + ey + f - gYx - hYy.$$

By adding in some zero terms, we get:

$$X = ax + by + c - 0d + 0e + 0f - gXx - hXy \ ,$$

and

$$Y = 0a + 0b + 0c + xd + ey + f - gYx - hYy.$$

Finally it becomes the product of a matrix and a vector:

$$\begin{bmatrix} x_1 & y_1 & 1 & 0 & 0 & 0 & -X_1x_1 & -X_1y_1 \\ 0 & 0 & 0 & x_1 & y_1 & 1 & -Y_1x_1 & -Y_1y_1 \\ x_2 & y_2 & 1 & 0 & 0 & 0 & -X_2x_2 & -X_2y_2 \\ 0 & 0 & 0 & x_2 & y_2 & 1 & -Y_2x_2 & -Y_2y_2 \\ \vdots & \vdots & \vdots & \vdots & \vdots & \vdots & \vdots & \vdots \\ x_n & y_n & 1 & 0 & 0 & 0 & -X_nx_n & -X_ny_n \\ 0 & 0 & 0 & x_n & y_n & 1 & -Y_nx_n & -Y_ny_n \end{bmatrix} \begin{bmatrix} a \\ b \\ c \\ d \\ e \\ f \\ g \\ h \end{bmatrix} = \begin{bmatrix} X_1 \\ Y_1 \\ X_2 \\ Y_2 \\ \vdots \\ X_n \\ Y_n \end{bmatrix}.$$

The vector (a,b,c,d,e,f,g,h) is then obtained by multiplying $(X_1,Y_1,X_2,Y_2,\cdots X_n,Y_n)$

with the inverse of $\begin{bmatrix} x_1 & y_1 & 1 & 0 & 0 & 0 & -X_1x_1 & -X_1y_1 \\ 0 & 0 & 0 & x_1 & y_1 & 1 & -Y_1x_1 & -Y_1y_1 \\ x_2 & y_2 & 1 & 0 & 0 & 0 & -X_2x_2 & -X_2y_2 \\ 0 & 0 & 0 & x_2 & y_2 & 1 & -Y_2x_2 & -Y_2y_2 \\ \vdots & \vdots & \vdots & \vdots & \vdots & \vdots & \vdots & \vdots \\ x_n & y_n & 1 & 0 & 0 & 0 & -X_nx_n & -X_ny_n \\ 0 & 0 & 0 & x_n & y_n & 1 & -Y_nx_n & -Y_ny_n \end{bmatrix}$, $n = 4$.

1. Criminisi A, Reid I, Zisserman A (1999) A plane measuring device. Image and Vision Computing 17: 625-634.
